# Chemical Suppression of Defects in Mitotic Spindle Assembly, Redox Control, and Sterol Biosynthesis by Hydroxyurea

**DOI:** 10.1534/g3.113.009100

**Published:** 2013-11-05

**Authors:** Andrew McCulley, Brian Haarer, Susan Viggiano, Joshua Karchin, Wenyi Feng

**Affiliations:** Department of Biochemistry and Molecular Biology, SUNY Upstate Medical University, Syracuse, New York 13210

**Keywords:** DNA replication, hydroxyurea, kinetochore-microtubule attachment, endoplasmic reticulum redox, ergosterol biosynthesis

## Abstract

We describe the results of a systematic search for a class of hitherto-overlooked chemical-genetic interactions in the *Saccharomyces cerevisiae* genome, which exists between a detrimental genetic mutation and a chemical/drug that can ameliorate, rather than exacerbate, that detriment. We refer to this type of interaction as “chemical suppression.” Our work was driven by the hypothesis that genome instability in a certain class of mutants could be alleviated by mild replication inhibition using chemicals/drugs. We queried a collection of conditionally lethal, *i.e.*, temperature-sensitive, alleles representing 40% of the yeast essential genes for those mutants whose growth defect can be suppressed by hydroxyurea (HU), known as a potent DNA replication inhibitor, at the restrictive temperature. Unexpectedly, we identified a number of mutants defective in diverse cellular pathways other than DNA replication. Here we report that HU suppresses selected mutants defective in the kinetochore-microtubule attachment pathway during mitotic chromosome segregation. HU also suppresses an *ero1-1* mutant defective for a thiol oxidase of the endoplasmic reticulum by providing oxidation equivalents. Finally, we report that HU suppresses an *erg26-1* mutant defective for a C-3 sterol dehydrogenase through regulating iron homeostasis and in turn impacting ergosterol biosynthesis. We further demonstrate that cells carrying the *erg26-1* mutation show an increased rate of mitochondrial DNA loss and delayed G1 to S phase transition. We conclude that systematic gathering of a compendium of “chemical suppression” of yeast mutants by genotoxic drugs will not only enable the identification of novel functions of both chemicals and genes, but also have profound implications in cautionary measures of anticancer intervention in humans.

A living organism’s genetic background determines how that organism interacts with the environment and, in humans, how we respond to medical intervention through drug therapies. Understanding how a given chemical interacts with a person’s genetic makeup is crucial for both achieving effective therapeutics and averting undesirable negative outcomes. Large-scale screens for chemical-genetic interactions have been exploited in nearly every model organism by the use of small molecules to reveal cellular pathways that respond to these molecules. Among them, the tractable organism *Saccharomyces cerevisiae* arguably represents the most expansive repertoire of mutants (Ben-Aroya *et al.* 2008; Li *et al.* 2011; Tong and Boone 2006; Winzeler *et al.* 1999), which has enabled the description of an array of interactions between a drug and a mutant. These interactions include those between a drug and the haploid gene deletion mutants, homozygous and heterozygous gene deletion mutants, as well as conditionally lethal mutants in the essential genes (Ben-Aroya *et al.* 2008; Giaever *et al.* 2004; Hillenmeyer *et al.* 2008; Hughes *et al.* 2000; Li *et al.* 2011; Lopez *et al.* 2008; Parsons *et al.* 2006). As the result of these studies we have gained a wealth of information regarding the chemical-genetic networks of a cell.

In nearly all of the aforementioned studies, much of the focus has been directed toward those chemical-genetic interactions that fall into one of two modes: “chemical sensitivity” or “chemical resistance” (Figure 1). Taken beyond the yeast genome, the former category is exemplified by the use of anticancer drugs to target the cancer genome, whereas the latter category is best demonstrated by the organismal response to antibiotics. Therefore, a drug is all too often regarded as an inhibitor of cellular functions that brings forth the demise of a given organism. Here we describe a third mode of chemical-genetic interaction, which has largely eluded researchers’ scrutiny, and our effort in systematic identification of these interactions. We sought those conditionally lethal mutations that can be *rescued*, rather than exacerbated, *by genotoxic drugs*. One likely reason why “chemical suppression” is rarely scrutinized might be because the interactions often are paradoxical, although they are far from being unprecedented in the literature. For instance, it was observed that azole fungal antibiotics could in fact *restore* viability in specific sterol auxotrophic mutants, which led to the discovery that the endogenously synthesized lanosterol can substitute for ergosterol (Gachotte *et al.* 1997). It was also reported that a temperature-sensitive mutant in the fission yeast Dam1/Dash outer kinetochore complex can be rescued by the antimicrotubule drug thiobendazole, demonstrating that the mutant suffers from hyperstabilized kinetochore-spindle malattachments (Griffiths *et al.* 2008). We also note that other published chemical-genetic screens often contain embedded “chemical suppression” data as well, although they are usually not the focus of the study (Li *et al.* 2011; van Pel *et al.* 2013).

**Figure 1:**
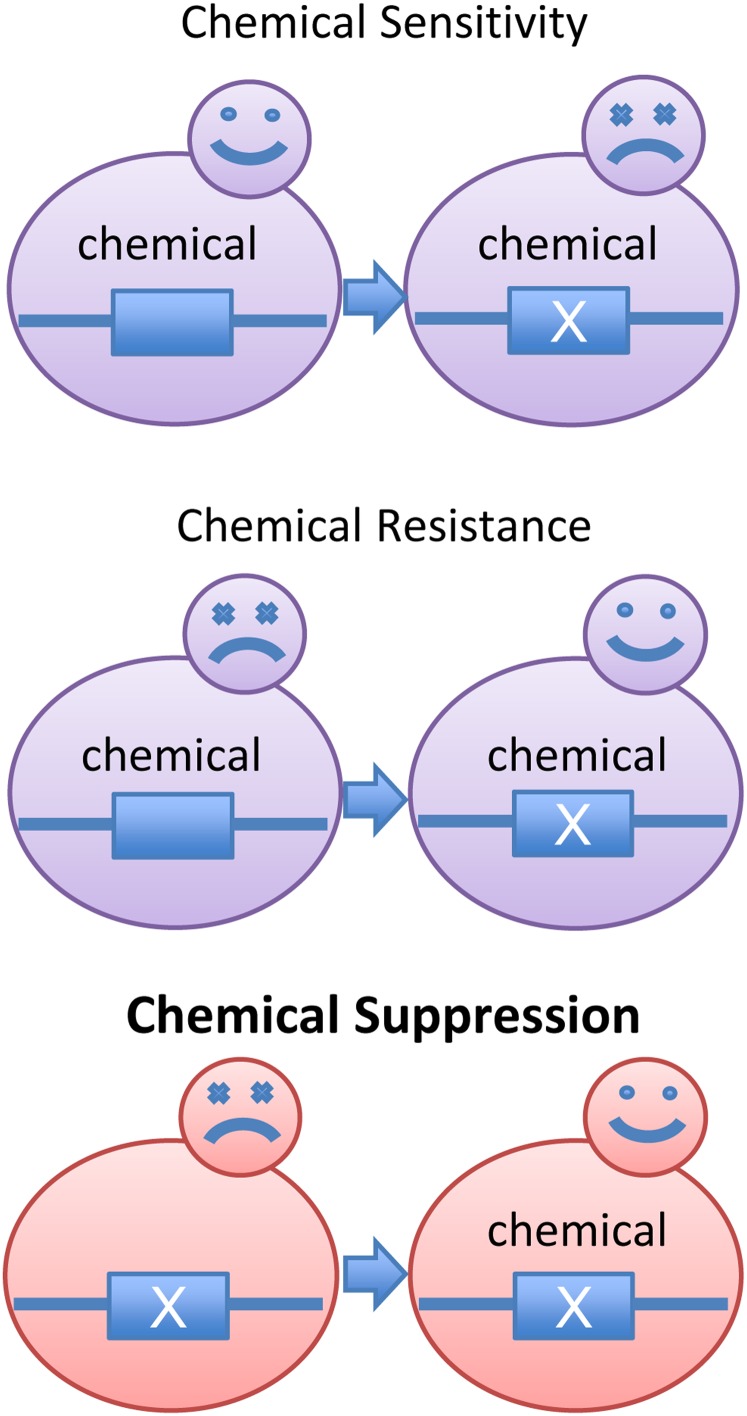
Schematic representation of three types of chemical-genetic interactions. “X” denotes a mutation in a given gene (blue box). Happy and sad faces indicate cell viability and lethality, respectively. In “chemical sensitivity,” cells without the mutation can survive chemical exposure, whereas cells carrying the mutation perish in the presence of the chemical. In “chemical resistance,” cells without the mutation perish in the presence of the chemical, whereas cells carrying the mutation are able to grow in the presence of the chemical. In “chemical suppression,” the roles of the mutation and the chemical are reversed from those in “chemical resistance” such that the cells carrying a lethal mutation can survive in the presence of the chemical.

Our work was inspired by the observation that the DNA replication inhibitor hydroxyurea (HU) can rescue a mutant carrying a temperature-sensitive allele (*ipl1-321*) of the Aurora B kinase, whose inactivation leads to defective chromosome segregation and genome instability (Figure 2) (Lampson and Cheeseman 2011). We referred to this type of interaction as “chemical suppression” and reasoned that there should be other mutants whose phenotypes are similarly alleviated by HU, either through its replication inhibitory function and/or other unknown functions. We note that “chemical suppression” is analogous to the suppression of a genetic mutation by another mutation, only with the latter’s role replaced by a chemical. We also note that “chemical suppression” is related to but different from “chemical resistance” (Figure 1), where the roles of the mutation and the drug are reversed. “Chemical suppression” has been described in pharmacological research where a drug/compound can act as an activator as opposed to an inhibitor. However, this specific class of “chemical suppression” is not likely informative of gene functions *per se* because the drug is often not well characterized and usually restores the function of a mutant protein structurally through direct binding. “Chemical suppression” has also been exploited in model organisms to screen a collection of small molecules for their capacity to reverse a specific phenotype due to genetic mutations (Baraban *et al.* 2013; Cao *et al.* 2009; Peal *et al.* 2011; Peterson *et al.* 2004; Su *et al.* 2010). However, in all these studies the screen was based on the concept of “many molecules *vs.* single phenotype.” To the best of our knowledge, a screen based on the “single molecule and many phenotypes” concept and to specifically catalog “suppression” events has not yet been conducted.

**Figure 2:**
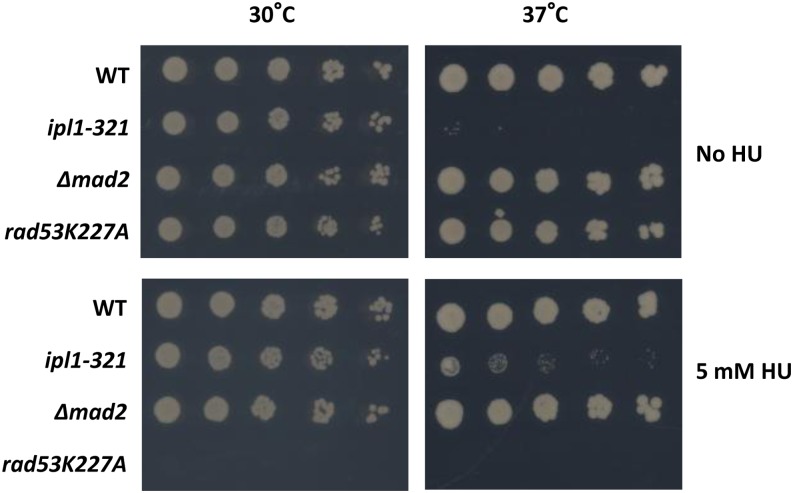
The TS of *ipl1-321* can be partially suppressed by 5 mM HU. Serial dilutions (1:5) of equal number of cells were spotted on YPD plates with or without 5 mM HU followed by incubation at the respective temperature for 3 d. The *Δmad2* strain is a positive control strain that is defective in the spindle checkpoint gene *mad2* but is not affected by HU. The *rad53K227A* strain is a negative control that is hypersensitive to HU.

We performed a “chemical suppression” screen by using a previously described collection of temperature-sensitive (TS) mutants (Li *et al.* 2011) and sought those mutants that showed improved growth in the presence of HU at the otherwise-restrictive temperature where the mutant would normally fail to grow. We hypothesized that there exist other mutants that can be rescued by HU by benefiting from mild replication inhibition, such as overreplication mutants. We also reasoned that HU might suppress other mutants through its yet unidentified or underappreciated functions other than the canonical function as an inhibitor of ribonucleotide reductase. We found that HU rescues the conditional lethality of mutants defective in diverse cellular pathways, including chromosome segregation, *endoplasmic reticulum* (ER) redox homeostasis, sterol biosynthesis, and mitochondrial genome maintenance. We provide evidence that in addition to its known function as a replication inhibitor, HU can also act as an oxidizing agent on the ER membrane as well as a regulator of iron homeostasis. We speculate that the ability of HU to rescue some of these mutants stems from a combination of its function as a replication inhibitor and these previously less well known “moonlighting” functions. Our data underscore the importance of using novel approaches to reveal new functions of drugs, particularly genotoxic drugs that are used in chemotherapy, to gain a comprehensive understanding of their interaction with the genome. Such information regarding drug functions would help prevent administering drugs that inadvertently benefit the cancer genome by affording it a growth advantage. Using the evolutionarily conserved and tractable system of *S. cerevisiae*, we believe that systematic searches for “chemical suppression” between these anticancer drugs and the yeast genome not only provide valuable information directly relevant to human health but also facilitate discoveries of novel drug and gene functions.

## Materials and Methods

### Yeast strains and screen conditions

“Chemical suppression” screens were performed with the TS mutant collection (*MAT***a** *his3*Δ*1 leu2*Δ*0 met15*Δ*0 ura3*Δ*0 yfg*::*yfg^ts^-KanMX*) from Charlie Boone’s laboratory at the University of Toronto (Li *et al.* 2011). Cells were grown in YPD medium containing G418 (200 μg/mL) for the screens. For streaking and spotting tests on YPD medium or YPD containing G418, either *BY4741* (*MAT***a** *his3*Δ*1 leu2*Δ*0 met15*Δ*0 ura3*Δ*0*) or a derivative of *BY4741* (*MAT***a** *YDR029w*::*KanMX his3*Δ*1 leu2*Δ*0 met15*Δ*0 ura3*Δ*0*), which contains the replacement of a dubious ORF with the *KanMX* marker, was used as the wild-type (WT) control, respectively. HU was added to a final concentration of 2.5 mM, 10 mM, 50 mM, or 100 mM. The TS collection was transferred to solid media in Omni plates using a Virtek Pinning Robot. The plates were photographed with a Canon EOS Rebel XTi 400D camera, and the images were manipulated and overlaid using GIMP or Photoshop. Petri dishes were photographed with a ScanMaker 9800XL Microtek scanner.

### Serial dilutions and spotting assay

Cell cultures were grown to a density of ~5 × 10^6^ to 10^7^ cells/mL and 1:5 serial dilutions were prepared in “-N” medium (1.61 g/L YNB without (NH_4_)_2_SO_4_ and amino acids, 94 mM succinic acid, and 167 mM NaOH) in 96-well plates. Equal volumes of cells (2 μL) were spotted on solid YPD medium containing 5, 10, 50, or 100 mM HU as indicated. Plates were incubated at 25°, 30°, or 37° and photographed after 2−3 d.

### Construction of *erg26-1* and *ero1-1* mutant strains *de novo* and Sanger sequencing

The introduction of *erg26-1* and *ero1-1* alleles into W303 strain background was performed using a strategy described previously (Li *et al.* 2011). To summarize, *erg26-1* and *ero1-1* loci were amplified by polymerase chain reaction (PCR) from the original isolates in the TS collection and linked to the *KanMX* marker via nested PCR. The resulting PCR products were used to transform a W303 homozygous diploid strain (*MAT***a/α** *ade2/ade2leu2/leu2his3/his3ura3/ura3trp1/trp1*). G418-resistant colonies were selected, and cells were sporulated followed by tetrad dissection on YPD medium. The *KanMX* marker segregated 2:2 as expected. Haploid progeny that were temperature-sensitive at 37° were identified. Three independent isolates of *erg26-1* strains (AMY1001-6A, AMY1006-7B, and AMY1010-8B; *MAT***a**) and two isolates of *ero1-1* strains (SCVY11 and SCVY13; *MAT***a**) were selected for further analysis. As controls, the *KanMX* marker was integrated adjacent to the WT *ERG26* and *ERO1* loci of the W303 background via identical strategy, resulting in AMY1013-1A and SCVY15-2D, respectively. The PCR and sequencing primers are listed in Supporting Information, Table S1. Sequence analysis was performed with ClustalW.

### Construction of a *cdc21Δ* strain carrying HSV-TK and hENT1 and in combination with the TS alleles of “chemical suppression” candidates

A *cdc21∆*::*KanMX/CDC21* (*MATa/MATα cdc21*::*KanMX4/CDC21leu2Δ0/leu2Δ0 his3Δ1/his3Δ1 ura3Δ0/ura3Δ0 lys2Δ0/LYS2met15Δ0/MET15*) strain from the Euroscarf (web.uni-frankfurt.de/fb15/mikro/euroscarf/) heterozygous diploid collection was converted to *cdc21∆*::*NAT/CDC21* by transformation of a purified *NAT* gene (encoding Nc acetyltransferase, which confers resistance to the antibiotic Nc, nourseothricin) from *Eco*RI digestion of plasmid p4339 (provided by C. Boone). The GPD^pr^-HSV-TK/*ADH1*^pr^-hENT-*LEU2* (“BrdU-Inc”) cassette was integrated into the aforementioned strain by transforming with *Pac*I-digested p405-BrdU-Inc plasmid (Viggiani and Aparicio 2006) and selecting for leucine prototrophy. [Note that Viggiani and Aparicio performed integration of *Hpa*I-digested p405-BrdU-Inc at the *leu2-3,112* mutant loci (Viggiani and Aparicio 2006), but this strategy is not feasible for the BY4743/Euroscarf strains containing the *leu2∆0* allele. Instead, the p405-BrdU-Inc plasmid was digested at the unique *Pac*I site and its integration was directed to a locus within the promoter region of the *ADH1* gene without disrupting the native *ADH1* function.] The resulting diploid strain was sporulated and tetrads were dissected on YPD medium supplemented with thymidine ranging from 1 mM to 4 mM, all of which supported the growth of the resulting *cdc21Δ*::*NAT ADH1*::*GPD^pr^-HSV-TK/ADH1^pr^-hENT-LEU2* segregants, as identified by screening for Nc resistance and leucine prototrophy. A *MAT*α segregant from these dissections was mated with *MAT***a** strains carrying relevant *KanMX4*-marked TS alleles of the “chemical suppression” candidates (*ipl1-1*, *ipl1-2*, *spc105-4*, *spc105-15*, *tub4-Y445D*, *erg26-1*, and *ero1-1*) and zygotes were isolated by either micromanipulation or by double selection on YPD medium containing 200 μg/mL G418 and 100 μg/mL nourseothricin. These diploids were subsequently sporulated and dissected to generate the desired segregants (by following three markers, *NAT*, *LEU2*, and *KanMX4*) carrying *cdc21∆*, BrdU-Inc and the aforementioned TS mutations, respectively. All resulting triple marked strains, along with all control strains, were grown overnight in liquid YPD medium supplemented with 4 mM thymidine at the permissive temperature, 25°. Cells were then diluted to the same density using fresh medium, followed by 1:5 serial dilution in “-N” medium. Equal numbers of cells (2 μL from each dilution) were spotted onto YPD, YPD containing 0.5 mM, 1 mM, 2 mM, and 4 mM thymidine, and YPD containing 4 mM thymidine and 10 mM HU, and incubated at 25°, 30°, 34.5°, and 37°. The plates were photographed after 2−3 d.

### Pulse field gel electrophoresis and Southern analysis

Pulse-field gel electrophoresis analysis was performed as described previously (van Brabant *et al.* 2001). Electrophoresis was conducted at 14° for 26 hr with a switch time ramped from 60 to 120 sec at 200 volts. Southern analysis was performed according to standard procedures.

### Petite frequency measurement

*ERG26* control cells (AMY1013-1A) and *erg26-1* cells (AMY1010-8B) were inoculated from a single colony into 5 mL of YPD medium, and the cultures were incubated at 25° for 24 hr. Cells were then diluted 1:1000 in fresh YPD medium with and without 10 mM HU, and each culture was then split into two aliquots and incubated at 25° or 30°, respectively, for another 24 hr. Cells were plated after sonication on solid YPD medium and the plates were incubated at 25°. Colonies were counted and the color recorded after 3−4 d. *Petite* frequency was calculated as the percentage of small white colonies that are not able to respire on YP medium containing 3% glycerol as a carbon source.

### Cell-cycle synchronization and flow cytometric analysis

Cells were grown in YPD liquid medium at 25° until they reached an OD_600_ of approximately 0.2. The cultures were then treated with α-factor (3 μM or 200 nM for *BAR1* or *bar1* strains, respectively) to synchronize the cell cycle. After removing α-factor by pronase (0.3 μg/mL) degradation each culture was split into two and incubated at 25° and 32°, respectively, for 15 min to equilibrate to the respective temperature. Samples were collected every 10 min for 90 min and fixed with ethanol. The samples were then stained with SYTOX Green and DNA content was measured with a BD LSR Fortessa. Data analysis was performed with FlowJo.

## Results

**Figure 3:**
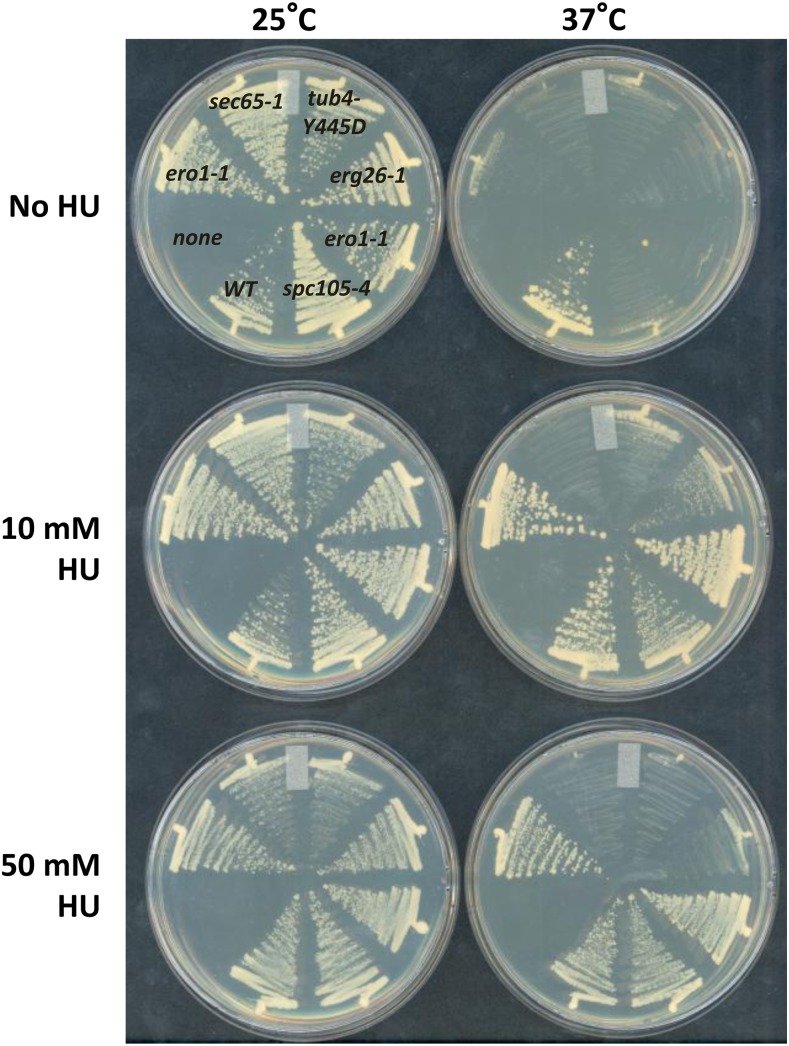
Confirmed “chemical suppression” by HU. Cells were streaked on plates containing no HU, 10 mM or 50 mM HU and the plates were incubated at 25° or 37° for 3 d before photographing. The arrangement of strains on each plate is as shown.

We queried a previously described collection of ~450 yeast TS mutants that represent approximately 40% of the essential genes in the yeast genome (Li *et al.* 2011). Each TS allele had been introduced into the BY4741 strain background using a *KanMX* marker (Li *et al.* 2011). We reasoned that the mutation spectrum that exhibits suppression by HU is likely dependent on drug concentration; therefore, we applied HU at 2.5, 10, 50, and 100 mM and sought those mutants that showed significant growth advantage in the presence of HU compared with that in the absence of HU at the restrictive temperature, 37°. We visually inspected overlaid images (see *Materials and Methods* for details) to identify such mutants, only accepting positive scoring if a given mutant was independently identified by at least two experimentalists. An example of the overlaid images of cells growing in the presence and absence of HU at the permissive and restrictive temperature is shown in Figure S1. Fifty-four primary candidates representing mutant alleles in 46 unique genes were subjected to a secondary screen by streaking or spotting serially diluted cells on solid media. Because only one mutant, *ero1-1*, was identified as a potential candidate at 2.5 and 100 mM HU, we decided to perform the secondary screen at 10 mM and 50 mM HU (Table 1 and Figure 3). Four of the candidates from the primary screen, *ero1-1*, *erg26-1*, *tub4-Y445D*, and *spc105-4*, successfully passed the secondary screen and showed improved growth in the presence of HU at 37° (Figure 3). We also confirmed that *ipl1-2* and *spc105-15*, which exhibit temperature sensitivity at 32° and 35°, respectively, were also suppressed by 10 mM HU at restrictive temperatures (data not shown). One mutant, *ero1-1*, was identified at all concentrations of HU. The other mutants showed varying levels of suppression by different concentrations of HU (Table 1). Although these mutants are defective in diverse cellular pathways, we demonstrate below that they fall into three functional groups, each of which interacts with HU through a distinct and previously unearthed function of the drug.

**Table 1:** List of mutants that are suppressed by HU as ascertained by secondary test of streaking or spotting on media containing 10 or 50 mM HU

Mutation	Temperature (°)	10 mM HU	50 mM HU
*ero1-1*	37	++++	++++
*erg26-1*	37	++	+
*spc105-4*	37	+++	+++
*spc105-15*	35	++	ND
*tub4-Y445D*	37	+	−
*ipl1-1*	35	+	−
*ipl1-2*	32	+	−

Increasing number of “+” indicates better suppression. “−”, no suppression observed. HU, hydroxyurea; ND, not determined.

### HU suppresses a group of mutants defective in bipolar chromosome attachment at the mitotic spindle, partially through delaying the cell cycle

*TUB4* encodes a ϒ-tubulin−related protein that nucleates microtubules at the spindle pole body (Marschall *et al.* 1996). Spc105, partnered with Kre28, forms one of the three complexes that link centromeric heterochromatin to kinetochore microtubules (Pagliuca *et al.* 2009). Ipl1 is responsible for correcting erroneous kinetochore-microtubule attachments, as reviewed by (Lampson and Cheeseman 2011). Tub4 is important for organizing the mitotic spindle as *tub4* mutants show excessive growth of microtubules (Marschall *et al.* 1996; Vogel *et al.* 2001). Effectively, mutations in all these genes lead to defective kinetochore-microtubule attachment and the mutants exhibit elevated frequency of mono-polar spindles (Lampson and Cheeseman 2011; Marschall *et al.* 1996; Pagliuca *et al.* 2009). These observations suggest that HU might suppress a general defect in kinetochore-microtubule attachment, but not kinetochore assembly *per se* as our primary screen did not identify any of the mutants in the inner or central kinetochore components. Consistent with this idea, selected mutants in the inner and central kinetochore components (*spc29*, *spc42*, *spc110*, *spc24*, and *spc25*) or the outer kinetochore components (*spc34* and *ndc80*) were not suppressed by either 10 or 50 mM HU (data not shown). In fact, a strain carrying the *ndc80* allele *tid3-1* is hypersensitive to 10 mM HU at even the permissive temperature (Table S2).

One trivial explanation is that HU, by delaying the cell cycle, allows sufficient time for proper kinetochore-microtubule attachment even though the suppression appeared specific as none of the other mutants in the chromosome segregation pathway in the TS collection was suppressed by HU. Nevertheless, we tested whether the temperature-sensitivity of these mutants could be alleviated by (1) nutrient-limited growth media, *i.e.*, those containing a sugar source that was either inefficiently used or at a lower than normal level; (2) inducing the replication checkpoint by overproducing the Rad53 kinase, and most importantly, (3) limiting exogenously supplied thymidine through the use of a *cdc21* mutant to mimic the nucleotide pool reduction effect of HU. First, we compared cell growth at the restrictive temperature on rich media (YP) containing 2% glucose to that on nutrient-limited media: YP containing 0.1% glucose, or 3% glycerol, or 3% ethanol, or 2% galactose and synthetic medium (SC) containing 2% glucose. Although the majority of the tested mutants, including *spc105-4*, *ipl1-1*, *ipl1-2*, *erg26-1*, and *ero1-1*, did not show improved growth at the restrictive temperature on nutrient-limited media, we did observe moderate suppression of the TS phenotype of *spc105-15* and *tub4-Y445D* on YP medium containing either 0.1% glucose or 2% galactose, as well as that of *tub4-Y445D* on SC medium with 2% glucose (Figure S2). These data indicate that merely slowing down the cell cycle was not sufficient to confer suppression of the majority of these TS mutants, but they do suggest that the *spc105-15* and *tub4-Y445D* mutants could partially benefit from the reduction of growth rate. Because cell-cycle delay triggered by HU is mediated through the Mec1/Rad53 kinase cascade, we also tested whether selected TS mutants that showed apparent specificity with HU (*ipl1-2* and *spc105-4*) can be suppressed by the overproduction of Rad53 through the use of a plasmid bearing *RAD53* under the *GAL1* promoter (J. Bachant, unpublished). We did not observe any suppression of the temperature-sensitivity of these mutants in growth medium containing either glucose (repressed for *GAL1* expression) or galactose (induced for *GAL1* expression; Figure S2).

Finally, we also tested whether delaying S-phase progression by limiting the nucleotide supplies contributed to the beneficial effects of HU. We took advantage of a previously described *cdc21Δ* mutant missing thymidylate synthase and relying on a reconstituted thymidine salvage pathway for survival (Vernis *et al.* 2003). We constructed such a *cdc21Δ* mutant with the ability to uptake thymidine from the growth medium by integrating constitutively expressed Herpes simplex virus thymidine kinase (HSV-TK) and human equilibrative nucleoside transporter (hENT1) (Viggiani and Aparicio 2006). We then constructed double mutants combining *cdc21Δ* expressing HSV-TK and hENT1 with each of the TS alleles: *spc105-4*, *spc105-15*, *ipl1-1*, *ipl1-2*, *tub4-Y445D*, *erg26-1*, and *ero1-1*. We examined whether the resulting double mutants showed improved growth, compared with the TS mutants alone, at the respective restrictive temperatures. As shown in Figure S3, for the majority of the mutants we did not observe improved growth of the double mutants relative to the TS mutants alone at any of the temperatures on media containing any amount of thymidine ranging from 500 μM to 4 mM. However, we did observe a mild suppression of the temperature-sensitivity of *spc105-4* by *cdc21Δ* in the presence of 4 mM thymidine, although not as strong as that achieved by HU (Figure S3). Therefore, we concluded that HU suppresses selected mutants in the mitotic spindle assembly pathway, including the *ipl1* mutants and, to a lesser degree and less specifically, the *spc105* mutants. In addition, HU specifically suppresses the *erg26* and *ero1* mutants neither through nutrient limitation nor through delaying S-phase progression, suggesting existent alternative function(s) of HU.

### HU suppresses *ero1-1* by providing an oxidation equivalent

**Figure 4:**
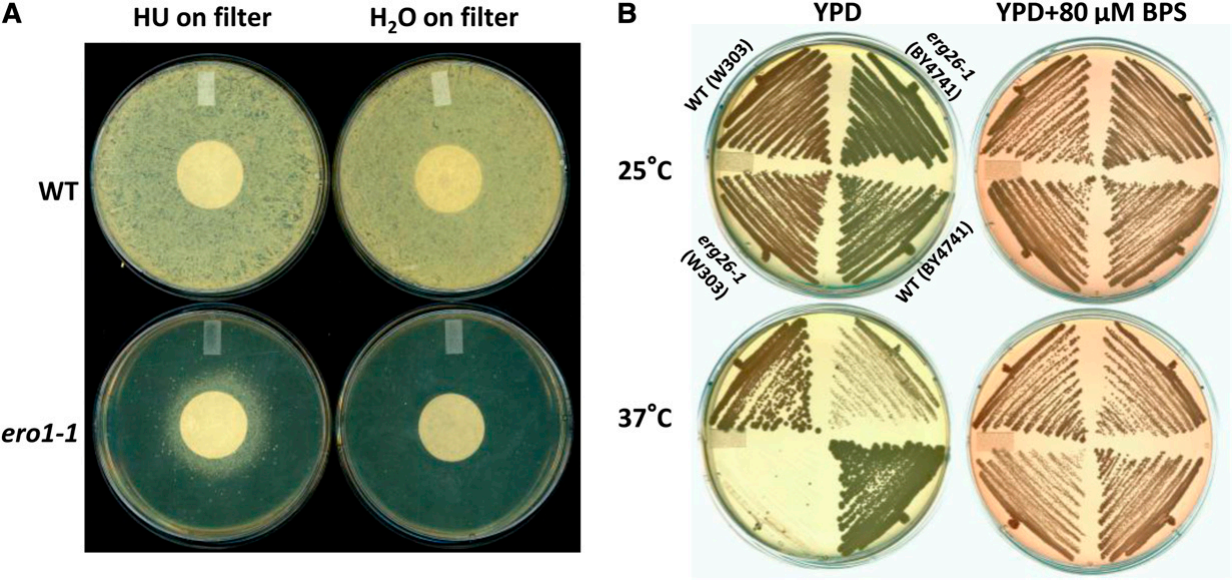
Proposed mechanisms of HU suppression of *ero1-1* and *erg26-1*. (A) HU can counteract the inhibitory effect of DTT on *ero1-1* cell growth. WT and *ero1-1* cells were spread on YPD plates containing 10 mM DTT. Equal volumes of H_2_O or HU (100 μmol) were spotted on the filter in the middle of the plate. Cells were grown at 30°C for two days before photographing. (B) The TS of *erg26-1* cells can also be suppressed by the iron chelator BPS. The original *erg26-1* mutant from the TS collection, *erg26-1* (BY4741), and a *de novo* introduction of *erg26-1* into the W303 background, *erg26-1* (W303), and their isogenic WT controls were streaked on YPD plates with or without 80 μM BPS. The positions of each strain on the four plates are identical and as indicated. Plates were incubated at 25° or 37° for 3 d before photographing.

The *ERO1* gene encodes a thiol oxidase that is essential for disulfide bond formation and protein folding in the ER (Frand and Kaiser 1998; Gross *et al.* 2006; Pollard *et al.* 1998). Ero1 acts as a supplier of the oxidative equivalents and transfers them to protein disulfide-bridge isomerase (PDI) via the oxidation of one of its three cysteine pairs. PDI can then bind unfolded proteins and help them fold by oxidizing structurally important disulfide bridges in these proteins. Thus, it is critical for cells to maintain an oxidizing environment in the ER to ensure proper function of PDI. We sequenced the *ero1-1* strain and confirmed that it contains a previously reported G229S mutation (Gross *et al.* 2004). The G229S mutation destabilizes the binding of Ero1 to a flavin cofactor at the restrictive temperature, which likely impairs its ability to oxidize PDI (Gross *et al.* 2004). However, if the oxidative state in the ER can be modified, then the Ero1 function can be bypassed. Consistent with this notion, temperature-sensitivity of *ero1-1* can be partially suppressed by diamide, an oxidizing agent (Frand and Kaiser 1998). Moreover, *ero1-1* cells are hypersensitive to the reductant dithiothreitol (DTT) (Frand and Kaiser 1998). Because HU is also an oxidizing agent, albeit a weak one, we wondered whether HU suppresses *ero1-1* via a similar mechanism as diamide. We used a filter test to examine this hypothesis. An equal number of cells (10^6^) were plated on solid YPD medium containing 10 mM DTT and bearing a filter disc in the middle. Either H_2_O or HU was spotted on the filter to allow the chemical to diffuse and form a gradient through the plate. If HU were able to suppress the lethality caused by DTT, a halo of cells surrounding the filter would appear. Indeed, we observed that *ero1-1* cells showed partial growth on the plate containing both DTT and HU (Figure 4A). Therefore, we concluded that HU suppresses *ero1-1*, at least partially, by oxidation.

### HU suppresses *erg26-1* by regulating iron homeostasis

*ERG26* encodes a C-3 sterol dehydrogenase in ergosterol biosynthesis and catalyzes the second of three steps in the removal of two methyl groups on 4,4-dimethylzymosterol to produce zymosterol (Baudry *et al.* 2001; Gachotte *et al.* 1998; Germann *et al.* 2005). We sequenced the *erg26-1* allele, previously unreported for the nature of the mutation, and found that it contains a G244D mutation resulting from a cytosine to thymidine change. We also serendipitously discovered that the *erg26-1* isolate exhibits an abnormal karyotype indicative of gross chromosomal rearrangement (Figure S4). Therefore, in order to confirm that the observed suppression by HU was specific to the *erg26-1* mutation, we introduced the *erg26-1* allele *de novo* into a WT strain of the W303 background. We confirmed that the newly created strains were temperature-sensitive, their suppression by HU was reproducible (Figure S5), and their karyotype was normal (data not shown). We also introduced the *erg26-1* allele *de novo* into a WT BY4741 strain and confirmed the TS phenotype and suppression by HU. However, the temperature-sensitivity is less penetrant in the BY4741 background than in the W303 background (data not shown). Thus, all subsequent analyses with *erg26-1*, unless otherwise noted, were performed with the W303 variant.

Because the action of the Erg26 enzyme also takes place in the ER (lipid bilayer), we considered the possibility that HU also suppresses *erg26-1* by redox control. However, unlike with *ero1-1*, *erg26-1* cells are not hypersensitive to DTT and their temperature-sensitivity cannot be suppressed by 0.6 mM diamide (Figure S6). We also note that the suppression of *erg26-1* is specific as HU does not suppress the other ergosterol mutants (*erg8-1*, *erg10-1*, and *erg11-td*) in the TS collection (Figure S7). In fact, the *erg10-1* strain, which contains a mutant form of the first enzyme in the ergosterol biosynthesis pathway, acetyl-CoA C-acetyltransferase, is hypersensitive to HU (Figure S7). Previously it has been shown that the lethal consequence of the *erg26-1* mutation is the accumulation of toxic zymosterol intermediates during ergosterol biosynthesis, rather than ergosterol deficiency (Baudry *et al.* 2001; Gachotte *et al.* 1998). Moreover, the temperature-sensitivity of an *erg26-1* strain can be suppressed by mutations in *ERG1*, *ERG7*, *ERG9*, *HEM1*, or *HEM3* (Baudry *et al.* 2001; Gachotte *et al.* 1998; Germann *et al.* 2005). The first three are all epistatic to *ERG26* in the ergosterol biosynthesis pathway and the latter two encode heme biosynthetic enzymes. These mutations all prevent the accumulation of the toxic zymosterol precursors. It has been shown that iron depletion negatively regulates both heme levels and sterol synthesis (Shakoury-Elizeh *et al.* 2010). It has also been reported that HU can cause up-regulation of the Aft1 iron regulon in yeast, suggesting that HU treatment causes intracellular iron deprivation (Dubacq *et al.* 2006). Therefore, we wondered whether HU suppresses *erg26-1 by* lowering intracellular iron levels. To test this hypothesis, we asked if *erg26-1* could also be suppressed by an iron chelator, bathophenanthroline disulfonate (BPS) (Yamaguchi-Iwai *et al.* 1995; Yamaguchi-Iwai *et al.* 1996). Indeed, temperature-sensitivity of *erg26-1* was rescued by BPS, as it was by HU (Figure 4B), consistent with the idea that lowering cellular iron level helps prevent the accumulation of toxic sterols. Thus, our data suggest that HU suppresses *erg26-1* through the regulation of iron homeostasis.

### *erg26-1* mutant shows elevated levels of *petite* formation

We were intrigued by the observation that the *erg26* strain from the TS collection exhibited abnormal karyotype and considered the possibility that the *erg26-1* mutation causes genome instability. Using our newly constructed *erg26-1* strain(s), we did not detect any gross chromosomal changes after growth of up to 100 generations at either the permissive (25°) or the semipermissive (30°) temperature (data not shown). Thus, the reason why the original *erg26-1* mutant in the Boone lab collection contained gross chromosomal rearrangements remains unknown. However, during the growth of the strains created in the W303 background we observed that although the WT cultures remained pink throughout the 100-generation growth, the *erg26* cultures appeared light pink or white (the W303 strain contains an *ade2* allele causing colonies to appear red due to the accumulation of an adenine biosynthesis intermediate, P-ribosylaminoimidazole, and either reversion/suppression of the *ade2* allele or the loss of the mitochondrial DNA genome can revert to the white colony phenotype). This observation prompted us to investigate whether the *erg26* mutant was producing *petites*—cells that have lost part or all of the mitochondrial genome—at a high frequency. Serial dilution and plating experiments showed that the *erg26* mutation did indeed result in an elevated *petite* frequency at both 25° and 30° (Table 2). Defective sterol synthesis was reported to influence mitochondrial membrane fluidity (McLean-Bowen and Parks 1982). We reasoned that altered membrane fluidity in the *erg26* mutant might incur damage to the mitochondrial membrane thereby causing mitochondrial DNA loss. We also observed that the increased *petite* frequency in the *erg26* mutant was suppressed by HU (Table 2).

**Table 2:** Percentage of *petite* colonies after 24 hr growth (approximately 12 generations) of *ERG26* and *erg26-1* cells at 25° and 30°, respectively, in two independent experiments

Experiment (culturing temperature)	*ERG26* (AMY1013-1A)		*erg26-1* (AMY1010-8B)	
	% *petites* (-HU)	% *petites* (+HU)	% *petites* (-HU)	% *petites* (+HU)
I (25°)	0.96 (n = 2594)	1.30 (n = 2532)	5.74 (n = 2089)	1.75 (n = 1604)
I (30°)	2.55 (n = 2788)	1.21 (n = 2487)	32.72 (n = 709)	10.47 (n = 993)
II (25°)	1.34 (n = 2456)	0.87 (n = 1502)	13.13 (n = 1766)	6.26 (n = 1518)
II (30°)	2.09 (n = 1870)	1.26 (n = 1673)	37.43 (n = 839)	27.02 (n = 359)

HU, hydroxyurea; n, total number of colonies counted.

### G1-to-S phase transition is delayed in the *erg26-1* mutant

**Figure 5:**
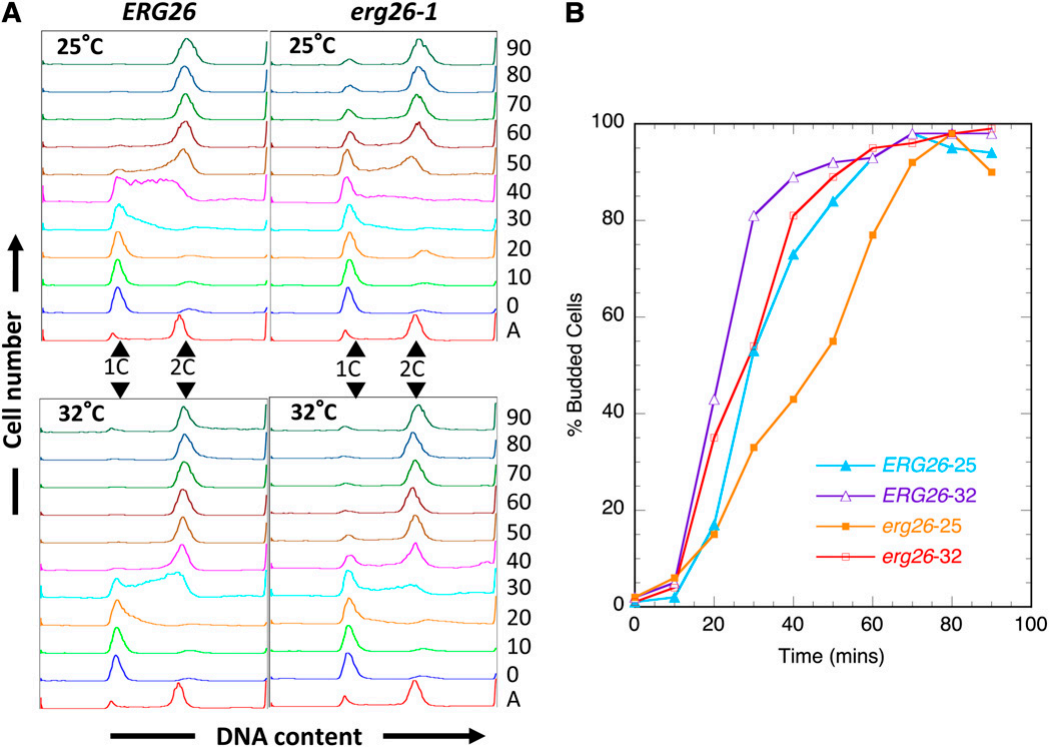
The *erg26* mutant is defective in G1-to-S transition. (A) Flow cytometric analysis of isogenic *ERG26* and *erg26-1* cells at 25°C or 32°C. Cells were synchronized by α-factor at the G1/S boundary (“0”), followed by release into S phase at the respective temperatures. Samples were collected every 10 min for 90 min “A,” asynchronous culture prior to G1/S arrest. (B) Budding indices for cells in (A). “−25” and “−32” indicate the culturing temperatures.

We also analyzed S-phase progression by flow cytometry in the *erg26-1* mutant to examine a potential defect in chromosomal DNA replication that might have caused the chromosome rearrangements in the original mutant strain. Cell cultures grown at 25° were synchronized with α factor at the G1/S boundary, split into two equal portions, then released into S phase at 25° and 32°, respectively. At 25°, *erg26* cells were delayed by at least 15 min in S-phase progression (Figure 5A). When cells are released at 32°, the entry into S phase was advanced in both *ERG26* and *erg26* cells, but the *erg26* cells still exhibited a mild delay in S-phase progression compared with the *ERG26* control (Figure 5A). To ascertain whether the delayed S-phase progression was attributable to a late entry into S phase and/or a reduced rate of DNA synthesis, we measured the budding index of these cells. We observed that the *erg26-1* mutant showed a delay in bud formation at both 25° and 32° (Figure 5B). Thus we concluded that the *erg26* mutant is defective in G1/S transition.

## Discussion

We have described a novel chemical-genetic screen to identify mutations whose defects can be suppressed by a DNA replication inhibitor, HU. Our initial goal was to unearth those novel genome stability mutations that cause either overreplication or untimely replication and therefore could benefit from mild replication inhibition by HU. Thus, we anticipated that a “chemical suppression” screen by using HU would identify mutations in genes that either directly participate in chromosomal DNA replication or its regulation. Instead, we identified a group of mutants (*spc105*, *ipl1*, and *tub4*) defective in bipolar kinetochore-microtubule attachment.

Because the common defect among the *tub4-Y445D*, *spc105-4*, and *ipl1-321* mutants is the lack of bipolar attachment of mitotic chromosomes (Biggins *et al.* 1999; Chan and Botstein 1993; Marschall *et al.* 1996; Pagliuca *et al.* 2009), we speculated that HU treatment might act at a step during kinetochore-microtubule (KT-MT) attachment that is specifically mediated by the Spc105 linker complex, which has multifaceted, yet specialized, functions at the kinetochore. The Spc105 linker complex is thought to stabilize the interaction between the spindle checkpoint proteins, such as Ipl1, and the kinetochore (Pagliuca *et al.* 2009). However, Spc105 is also thought to be crucial for recruiting the Glc7 phosphatase to the kinetochore to counterbalance Ipl1 kinase activity (Pinsky *et al.* 2009; Rosenberg *et al.* 2011). Therefore, Spc105 might control the opposing activities of Ipl1 (kinase) and Glc7 (phosphatase) at the kinetochore to ensure proper dynamics of the KT-MT attachment. Supporting this notion was the observation that mutations in the *GLC7* gene suppress a TS *ipl1-2* mutant (Tatchell *et al.* 2011). Interestingly, a recent study also showed that the temperature-sensitivity of the *ipl1-321* mutant could be partially suppressed by the microtubule-destabilizing drug, benomyl, suggesting that the *ipl1* mutant suffers from hyperstabilized KT-MT attachment (Cairo *et al.* 2013). Together these findings allow us to postulate that (1) the *spc105* mutant(s) also suffer from hyperstabilized KT-MT attachment; and (2) HU treatment destabilizes KT-MT attachment whereby it suppresses these KT-MT attachment mutants, possibly by modulating the Ipl1 kinase and Glc7 phosphatase activities at the KT. Whether and how HU modulates Spc105 function, or indeed the kinase and phosphatase activities at the kinetochore, either directly or indirectly, remains to be tested. Nevertheless, our findings might help delineate the functions of those genes that can be suppressed by HU from the functions of those that cannot, in the chromosome segregation pathway.

We also discovered other previously uncharacterized functions of HU on the ER membrane. We showed that HU could provide to the ER membrane the oxidation equivalent that is lacking in the *ero1-1* mutant and promote the oxidizing environment for protein disulfide bond formation and proper folding. HU suppresses *ero1-1* very robustly at concentrations ranging from 2.5 to 100 mM without apparent toxicity and the suppression is superior to that by diamide (Figure S6), so we also entertained the possibility that the suppression is multifaceted. Because it has been shown that HU can induce the oxidative stress response pathway mediated by Yap1 (Dubacq *et al.* 2006), HU might also suppress *ero1-1* by up-regulating Cu, Zn superoxide dismutase, which is required for the tolerance of ER stress (Tan *et al.* 2009). Moreover, the lack of Ero1p function triggers the unfolded protein response, which in turn regulates cell cycle progression (Rutkowski and Hegde 2010). For instance, the loss of unfolded protein response signaling can lead to chromosome loss events (Henry *et al.* 2010). Notably, *ero1-1* cells are defective in cytokinesis at the restrictive temperature 37°, but DNA replication and nuclear division are unhindered and the cells display a 3C or 4C DNA content and effectively accumulate aneuploidy (Bicknell *et al.* 2007). Therefore, it is possible that HU can suppress *ero1-1* not only by oxidation but also by preventing over-replication in the absence of cytokinesis. We asked whether *ero1* could be suppressed by other replication inhibitors such as methyl methanesulfonate (MMS, an alkylation agent). Interestingly, MMS can suppress the temperature-sensitivity of *ero1* cells at both 0.006% and 0.03%, albeit weakly when compared to HU (data not shown). A recent genome-wide study indicated that MMS elicits global cellular response to oxidative stress (Tkach *et al.* 2012); therefore, we surmised that MMS might also function as an oxidant and provide partial suppression of the *ero1-1* mutant. However, the robust suppression of *ero1-1* by HU likely stems from its unique and multifaceted functions that cannot be easily substituted by other replication inhibitors.

We also demonstrated that HU suppresses an *erg26-1* mutant defective in the ergosterol biosynthesis pathway. We presented evidence that this suppression is mediated through the regulation of iron homeostasis, which in turn regulates ergosterol biosynthesis and prevents the accumulation of toxic sterol intermediates. Though it was demonstrated that the *erg26-1* mutant suffers from accumulation of toxic sterol intermediates (Baudry *et al.* 2001; Gachotte *et al.* 1998), the mechanism of sterol toxicity leading to cell lethality remains unknown. Erg26 is one of the three enzymes—the other two being Erg25 and Erg27—that metabolizes 4,4-dimethylzymosterol (Bard *et al.* 1996; Gachotte *et al.* 1999; Li and Kaplan 1996). Yeast cells lacking functional Erg25 and Erg27 also have been shown to accumulate methyl sterols and methyl zymosterone, respectively, that are also potentially toxic sterols (Gachotte *et al.* 1999; Li and Kaplan 1996). If HU suppresses *erg26-1* through general inhibition of the sterol biosynthesis pathway, it would follow that HU would also suppress the *erg25* and *erg27* mutants (they are not present in our TS collection). However, Erg25 contains an oxy-diiron center, which lends itself to be sensitive to iron depletion (Li and Kaplan 1996). In fact, it has been shown that an *erg25* mutant that shows normal growth in iron-replete medium is hypersensitive to iron-limitation (Li and Kaplan 1996). Therefore, the *erg25* mutant, rather than being suppressed by, might actually be hypersensitive to HU. In contrast, Erg27 does not contain either an oxy-diiron center or an iron-sulfur cluster based on computational prediction and therefore the *erg27* mutant might be rescued by HU. During the preparation of this manuscript, we obtained an *erg27* TS mutant from Phillip Hieter’s lab and tested its potential rescue by HU at the restrictive temperature (Ben-Aroya *et al.* 2008). To our surprise the *erg27* mutant was hypersensitive to 10 mM HU at the permissive temperature and was not suppressed by HU at the restrictive temperature (data not shown). Therefore, we hypothesize that HU specifically inhibits the accumulation of toxic sterols, 4-carboxy-zymosterol and 4-methyl-4-carboxy-zymosterol, in the *erg26* mutant (Bard *et al.* 1996). It will be interesting to investigate whether HU directly affects the metabolism of these sterol intermediates.

During our characterization of the *erg26* mutant we discovered that the original *erg26-1* mutant from the Boone lab contains gross chromosomal rearrangement while all other ergosterol mutants in the same collection were apparently karyotypically normal. It is unclear what event(s) precipitated such genetic alterations in this strain and we have not been able to obtain clones containing gross chromosomal rearrangement after *de novo* introduction of the *erg26-1* mutation into a WT strain background. However, we observed increased *petite* frequency in the *erg26* mutant at both the permissive and semi-permissive temperatures and that this phenotype can be suppressed by HU. Defective sterol synthesis could have an impact on mitochondrial membrane fluidity (McLean-Bowen and Parks 1982). Moreover, mutants in the ergosterol biosynthesis pathway, including *erg26*, have been reported to show abnormal mitochondrial morphology (Altmann and Westermann 2005). Because the lethality of the *erg26* mutant at high temperature is linked to the accumulation of toxic sterols, we speculate that the incorporation of toxic sterols into the mitochondrial membrane could upset its sterol to lipid ratio and that HU can alleviate such damage to the mitochondrial membrane thereby reducing *petite* frequency. We also observed that the *erg26* mutant is delayed in the G1-to-S transition of the cell cycle. We surmise that even at the permissive temperature the *erg26* mutant is defective in the sterol composition of its cellular membranes and thus triggers a delay in the *START* of G1. Thus, it is conceivable that the *erg26* mutation might also alter the normal sterol composition of the nuclear envelope such that it might affect the interactions between the chromosomes and the nuclear envelope and/or the embedded spindle pole bodies, thereby perturbing chromosome segregation.

**Figure 6:**
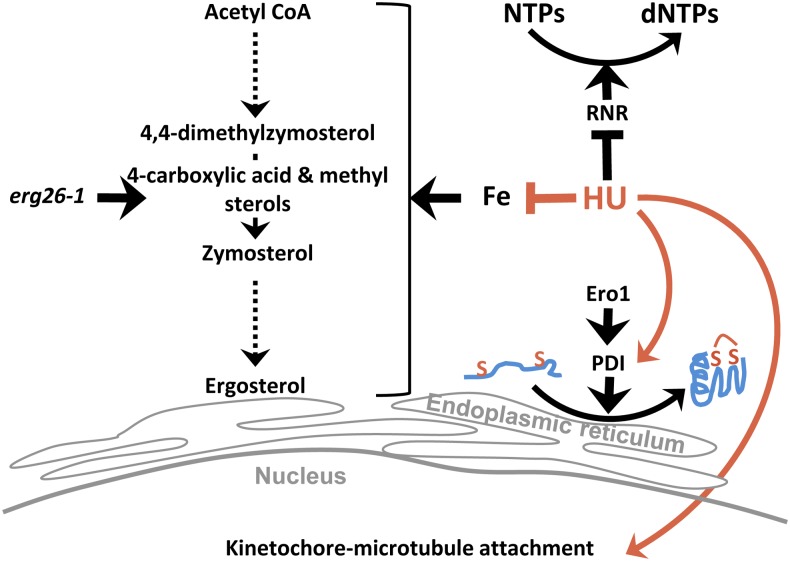
Summary of the multifaceted functions of HU. Note that both Erg26 and Ero1 function in the ER. dNTP, deoxyribonucleoside triphosphate; NTP, nucleoside triphosphate; RNR, ribonucleotide reductase. PDI, protein disulfide isomerase.

In summary, we have uncovered multifaceted functions of a small molecule previously widely regarded as a replication inhibitor (Figure 6). Our findings warrant reevaluation of mutations previously annotated to show either positive or negative genetic interactions with HU, which hitherto have been largely considered defective in the replication checkpoint pathway or DNA replication. Systematic identification of “chemical suppression” adds a new dimension to the known chemical-genetic interaction network. By mimicking a genetic mutation, chemicals and drugs can exert both negative and positive effects on an existent mutation. Thus, “chemical suppression” screens will help us devise cautionary measures in chemotherapy to avoid administering drugs that can suppress mutations in the cancer genome. Furthermore, “chemical suppression” screens have implications in the identification of novel functions and therapeutic potentials of existing chemical compounds. Such novel applications of existing drugs are well documented. For instance, the microtubule-stabilizing anticancer drug, paclitaxel, was found effective in the treatment of Alzheimer’s disease by breaking up tau protein aggregates in a mouse model (Zhang *et al.* 2005). Anticancer drugs have also been found as potent inhibitors of *Plasmodium falciparum*, the malaria parasite (Nzila *et al.* 2010).

We suggest that “chemical suppression” screens in yeast provide an efficient and meaningful tool for novel drug function discovery. The yeast ergosterol biosynthetic pathway shares similarities with cholesterol biosynthesis in humans. In fact, the murine and human homologs of *ERG26*, NAD(P)-dependent steroid dehydrogenase-like (NSDHL), have both been shown to complement yeast cells lacking *erg26* (Lucas *et al.* 2003; McLarren *et al.* 2010). Moreover, a recent study reported that mutations in *nsdhl* are linked to the CK syndrome, an X-linked recessive intellectual disability syndrome (McLarren *et al.* 2010), as well as the CHILD (congenital hemidysplasia with ichthyosiform nevus and limb defects) syndrome, an X-linked dominant, male-lethal disease (Konig *et al.* 2000). Significantly, analyses of male-lethal embryos in a mouse model of the CHILD syndrome suggest that male lethality is not accompanied by deficiency in either cholesterol or total sterol level (Caldas *et al.* 2005). Therefore, we are currently testing if HU also can promote cell viability in yeast cells carrying the mutant alleles of *nsdhl* containing SNPs found in the CHILD syndrome patients.

## Supplementary Material

Supporting Information
