# Supplementary material for: Chemical Suppression of Defects in Mitotic Spindle Assembly, Redox Control, and Sterol Biosynthesis by Hydroxyurea
Source: G3 (Bethesda). 2013 Nov 5;4(1):39–48. doi: 10.1534/g3.113.009100 (PMC3887538; doi:10.1534/g3.113.009100)
Supplement: Supporting Information [file supp_4_1_39__index.html]

Chemical Suppression of Defects in Mitotic Spindle Assembly, Redox Control, and Sterol Biosynthesis by Hydroxyurea — Supporting Information 

# Chemical Suppression of Defects in Mitotic Spindle Assembly, Redox Control, and Sterol Biosynthesis by Hydroxyurea

## Supporting Information for McCulley *et al.*, 2014

**Files in this Data Supplement:**

- Supporting Information - Figures S1-S7 and Tables S1-S2 (PDF, 2 MB)
- Figure S1 - Representative overlaid images of a primary "Chemical Suppression" screen. (PDF, 374 KB)
- Figure S2 - (A) The majority of the mutants whose temperature-sensitivity was rescued by HU cannot be rescued by nutrient limitation-induced cell cycle delay. (B) Overexpression of the Rad53 kinase does not rescue the temperature sensitivity of the mutants suppressed by HU. (PDF, 574 KB)
- Figure S3 - Thymidine limitation does not suppress most of the temperature-sensitive alleles that are suppressed by HU. (PDF, 1 MB)
- Figure S4 - The original *erg26-1* strain from the TS collection exhibits gross chromosomal rearrangement. (PDF, 402 KB)
- Figure S5 - *De novo* introduction of *erg26-1-KanMX4* into W303 strain. (PDF, 476 KB)
- Figure S6 - Temperature-sensitivity of *ero1-1* but not *erg26-1* cells can be partially suppressed by the oxidizing chemical diamide. (PDF, 460 KB)
- Figure S7 - HU specifically suppresses *erg26-1*, not other ergosterol mutants in the temperature-sensitive strain collection. (PDF, 497 KB)
- Table S1 - Sequence of PCR and sequencing primers for *ERO1* and *ERG26*. (PDF, 414 KB)
- Table S2 - List of mutations that show hypersensitivity to HU. (PDF, 391 KB)
