# Supplementary material for: Chemical Suppression of Defects in Mitotic Spindle Assembly, Redox Control, and Sterol Biosynthesis by Hydroxyurea
Source: G3 (Bethesda). 2013 Nov 5;4(1):39–48. doi: 10.1534/g3.113.009100 (PMC3887538; doi:10.1534/g3.113.009100)
Supplement: Supporting Information [file supp_g3.113.009100_009100SI.pdf]

G3·Genes | Genomes | Genetics

Chemical Suppression of Defects in Mitotic Spindle Assembly, Redox Control and Sterol Biosynthesis by Hydroxyurea

Andrew McCulley[§], Brian Haarer[§], Susan Viggiano, Joshua Karchin, and Wenyi Feng*

Department of Biochemistry and Molecular Biology
SUNY Upstate Medical University
750 East Adams Street
Syracuse, NY 13210
[§]Authors contributed equally to this work.
*Corresponding author: fengw@upstate.edu

DOI: 10.1534/g3.113.009100

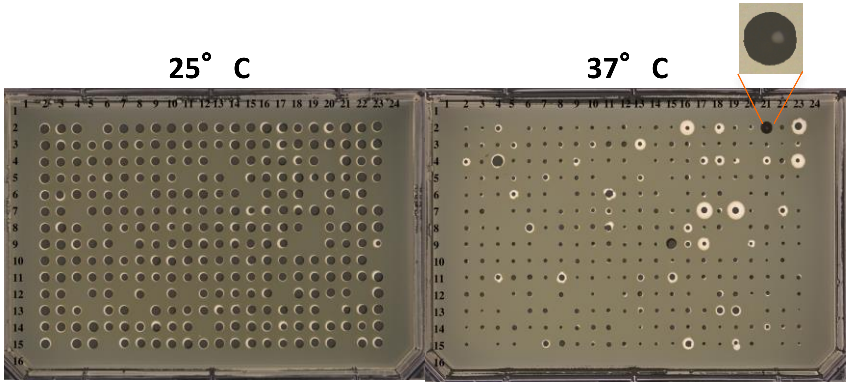

Figure S1 Representative overlaid images of a primary "Chemical Suppression" screen. Cells that were grown in the absence and presence of 50 mM HU were artificially colored white and black, respectively. The left and right images correspond to plates incubated at 25°C and 37°C, respectively. The majority of the temperature-sensitive mutant collection demonstrated temperature-sensitivity at 37°C while a small percentage showed either moderate or no temperature-sensitivity (comparing the white colonies at 25°C and 37°C). We believe that some of these strains might have accumulated suppressor mutations or are revertants. Those cells that showed enhanced growth in the presence of HU at 37°C appear as a grey spot with a black halo, such as *ero1-1* located at row 2, column 21 (inset). The plates were photographed after 3 days growth.

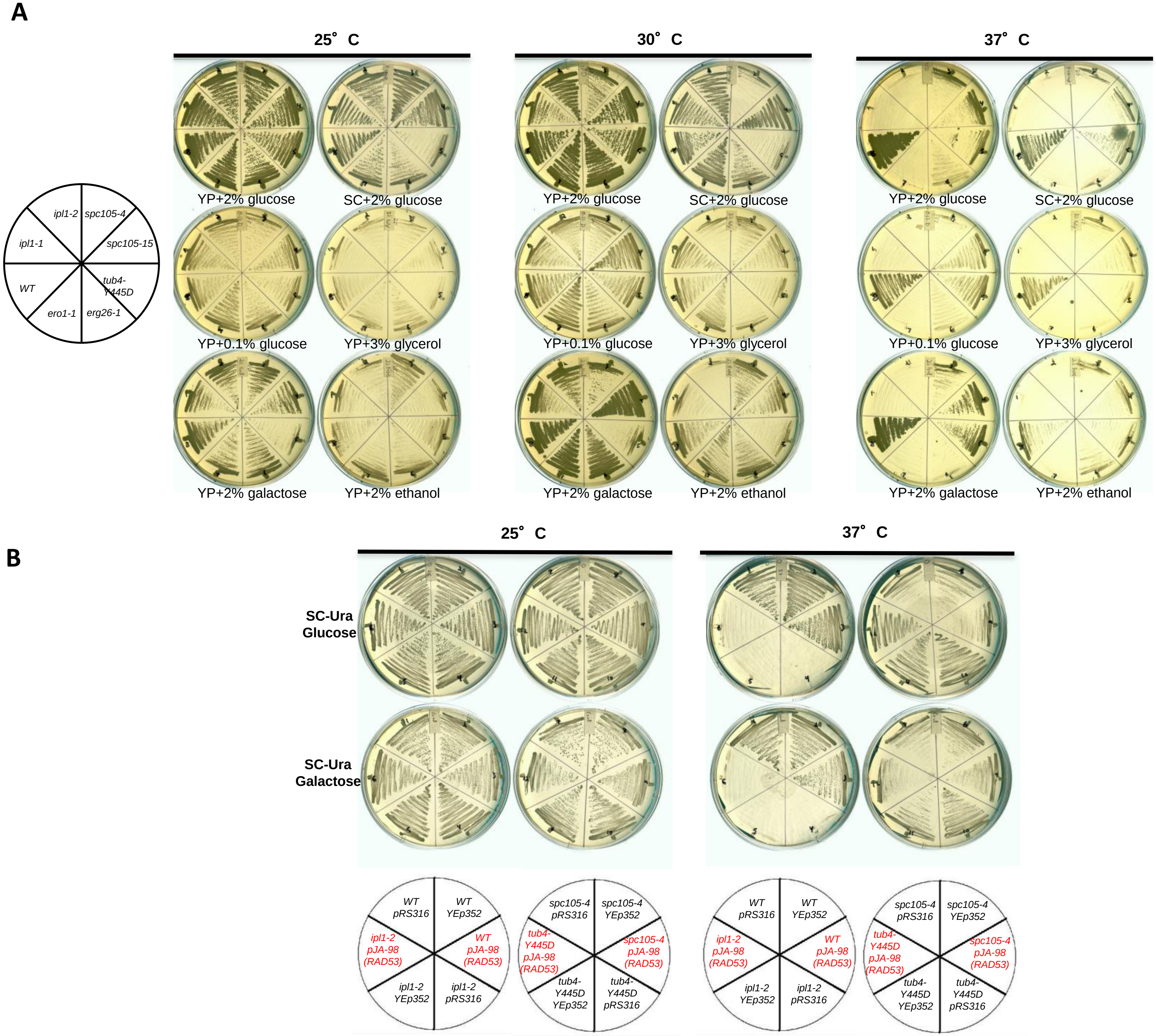

A
25° C
30° C
37° C
YP+2% glucose
SC+2% glucose
YP+0.1% glucose
YP+3% glycerol
YP+2% galactose
YP+2% ethanol
ipl1-2
spc105-4
ipl1-1
spc105-15
WT
tub4-Y445D
ero1-1
erg26-1
B
SC-Ura Glucose
SC-Ura Galactose
WT pRS316
WT YEp352
ipl1-2 pJA-98 (RAD53)
WT pJA-98 (RAD53)
ipl1-2 YEp352
ipl1-2 pRS316
spc105-4 pRS316
spc105-4 YEp352
tub4-Y445D pJA-98 (RAD53)
spc105-4 pJA-98 (RAD53)
tub4-Y445D YEp352
tub4-Y445D pRS316

Figure S2 (**A**) The majority of the mutants whose temperature-sensitivity was rescued by HU cannot be rescued by nutrient limitation-induced cell cycle delay. Growth media with different carbon sources, identical in each of the three panels under different temperatures, are as shown. Plates were photographed after three days incubation at the respective temperatures. The order of strains on each plate is indicated by the key on the left. (**B**) Overexpression of the Rad53 kinase does not rescue the temperature sensitivity of the mutants suppressed by HU. WT, *ipl1-2, spc105-4,* and *tub4-Y445D* strains were transformed with the pRS316, YEp352, or pJA-98 (GAL-RAD53) plasmids bearing *URA3* prototrophic marker. pRS316 and YEp352 are empty plasmid controls for the pJA-98(GAL-RAD53) plasmid. The resulting transformants were streaked on SC medium lacking uracil with either 2% glucose (repressed for *RAD53* expression) or 3% galactose (induced for *RAD53* expression). The plates were incubated at either 25°C or 37°C for two days before photographing. The keys to the strains on each plate are shown at the bottom of the panel.

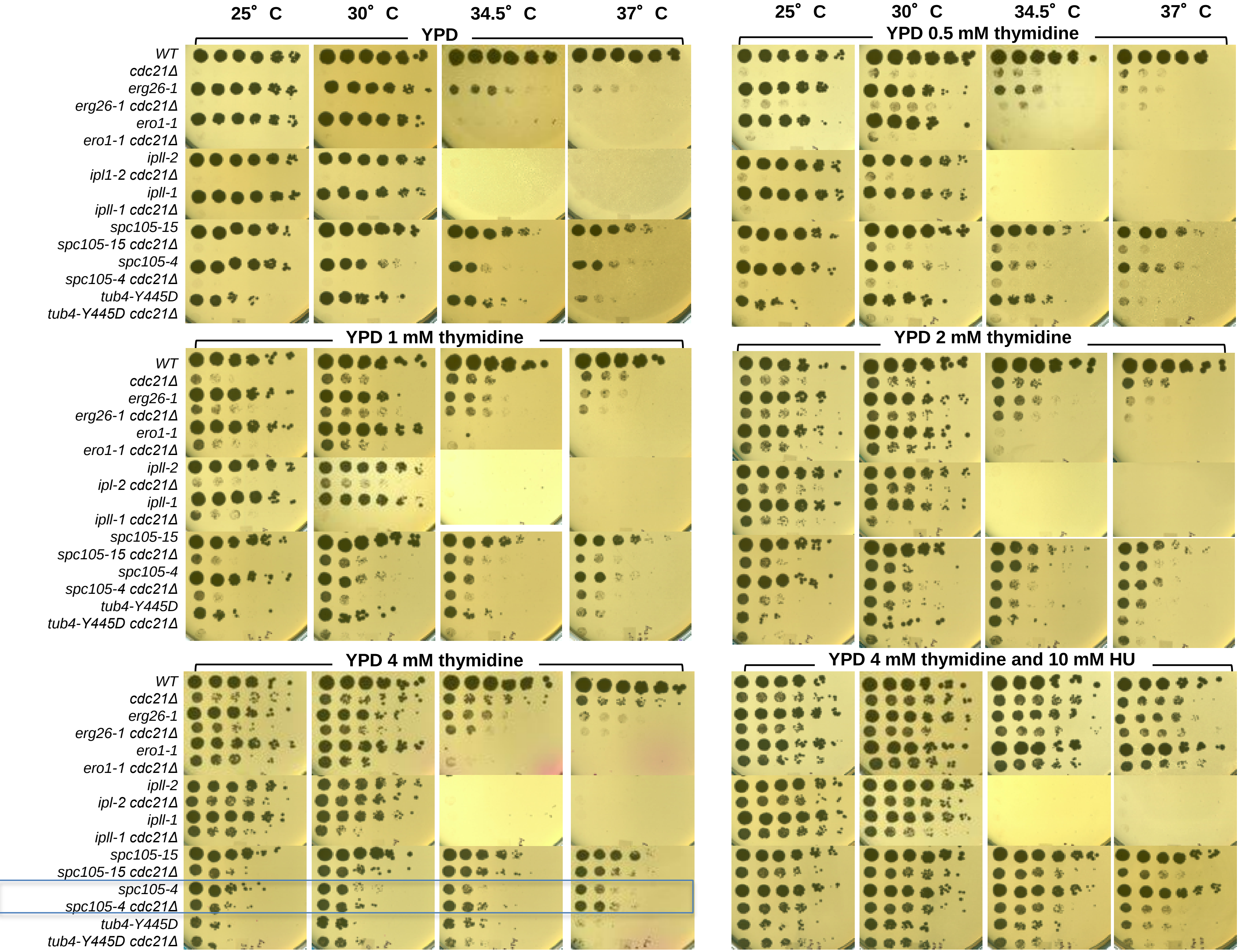
25° C
30° C
34.5° C
37° C
YPD
YPD 0.5 mM thymidine
YPD 1 mM thymidine
YPD 2 mM thymidine
YPD 4 mM thymidine
YPD 4 mM thymidine and 10 mM HU
WT
cdc21Δ
erg26-1
erg26-1 cdc21Δ
ero1-1
ero1-1 cdc21Δ
ipll-2
ipl1-2 cdc21Δ
ipll-1
ipll-1 cdc21Δ
spc105-15
spc105-15 cdc21Δ
spc105-4
spc105-4 cdc21Δ
tub4-Y445D
tub4-Y445D cdc21Δ
ipl-2 cdc21Δ

Figure S3 Thymidine limitation does not suppress most of the temperature-sensitive alleles that are suppressed by HU. Serial diluted cells were spotted on YPD media containing the indicated concentrations of thymidine and incubated at the indicated temperatures for 2-3 days before photographing. YPD medium containing 4 mM thymidine and 10 mM HU served as a control.

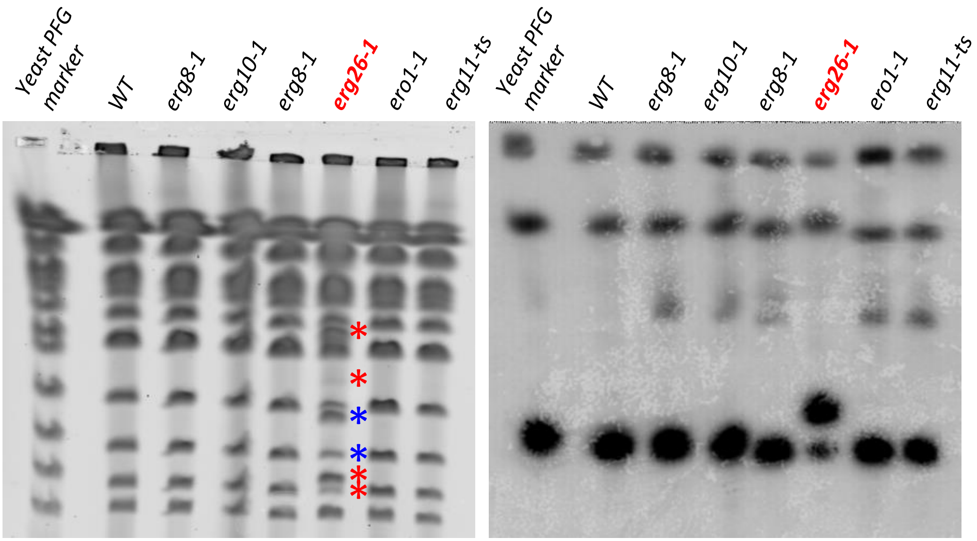

Figure S4 The original *erg26-1* strain from the TS collection exhibits gross chromosomal rearrangement. (**A**) Pulse Field Gel Electrophoresis of yeast chromosomes isolated from different temperature-sensitive strains in the collection. Those chromosome bands that show anomaly such as intensity gain, loss or different size from the control are marked by an asterisk. The blue asterisks indicate the chromosome III species recognized by a mixture of ARS301 and ARS319 probes in (B). (**B**) Southern hybridization of the gel in (A) using a mixture of ARS301 and ARS319 probes.

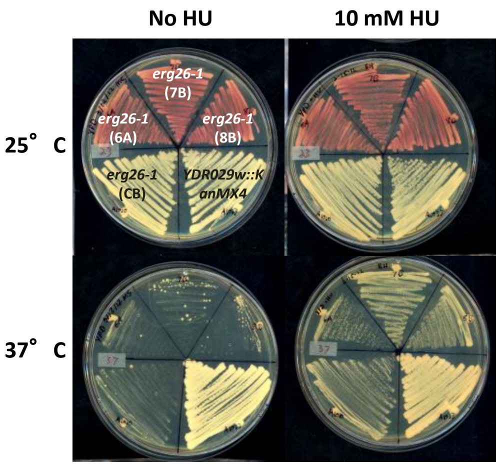

Figure S5 The three isolates of *erg26-1* in W303 (6A, 7B and 8B) are also temperature-sensitive at 37°C and the temperature-sensitivity can be suppressed by 10 mM HU. Cells were streaked on solid YPD medium containing 200 µg/ml G418 with or without HU. A strain containing the replacement of a dubious ORF YDR029w by the KanMX4 cassette and the *erg26-1* strain from the TS collection, *erg26-1* (CB), both in the BY4741 background, served as controls.

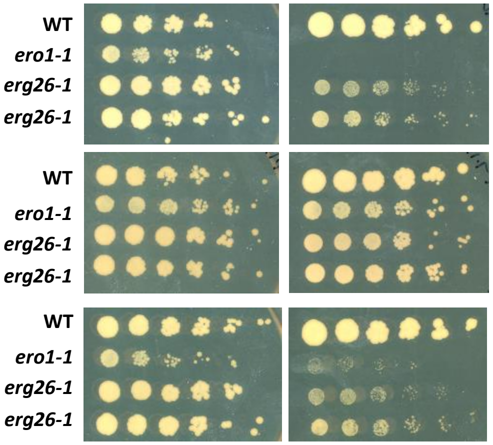

Figure S6 Temperature-sensitivity of *ero1-1* but not *erg26-1* cells can be partially suppressed by the oxidizing chemical diamide. Serial dilutions (1:5) of *ero1-1* and *erg26-1* cells were spotted on solid YPD media containing no drug, or 10 mM HU, or 0.6 mM diamide and incubated at the indicated temperatures for two to three days before photographing.

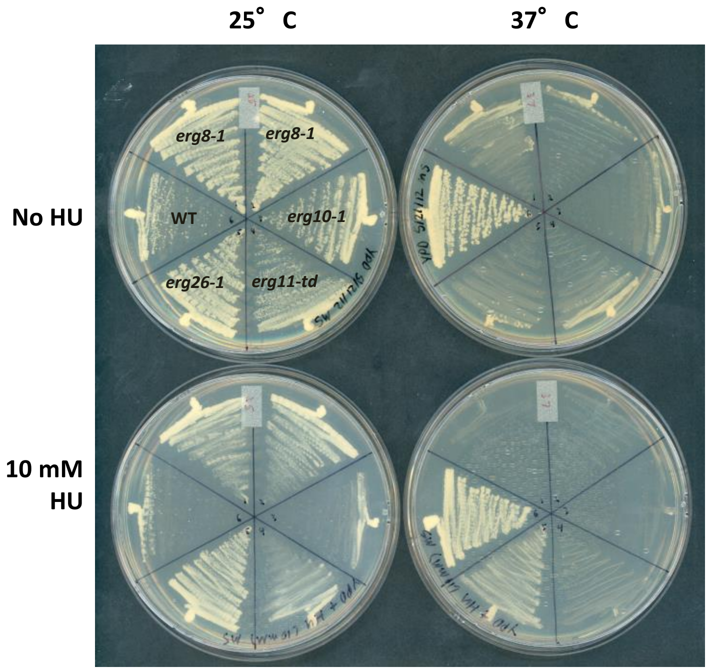

Figure S7 HU specifically suppresses *erg26-1,* not other ergosterol mutants in the temperature-sensitive strain collection. The indicated strains were streaked on YPD medium with or without 10 mM HU and incubated at the indicated temperatures for two to three days before photographing.

Table S1 Sequence of PCR and sequencing primers for *ERO1* and *ERG26*.

**PCR primers**	
ERG26-F1	5'-gcaactctaccggaagggcaaac-3'
ERG26-R2	5'-gacccggcggggacgaggcaagctaaacagatctattaccccgacgcttcatagtgtagtc-3'
ERO1-F1	5'-acgatacggagtacgtgtcataaaaacttg-3'
ERO1-R2	5'-gtgacccggcggggacgaggcaagctaaacagatctattgatatggaagggtctatttagcggtgt-3'
**Sequencing primers**	
ERG26-seq-R1	5'-cggtgcgtatcagatatgc-3'
ERG26-seq-R2	5'-ggacgaagagcaacagtatag-3'
ERO1-seq-F1	5'-tatggtggtaagcaagctgg-3'
ERO1-seq-F2	5'-caacgacgctgatgaattc-3'
ERO1-seq-R2	5'-gtttgtcactctatcagg-3'

Table S2 List of mutations that show hypersensitivity to HU.

**10 mM HU**	
**Mutation**	**Gene function**
prp9-ts	Subunit of the SF3a splicing factor complex, required for spliceosome assembly; acts after the formation of the U1 snRNP-pre-mRNA complex
sup35-td	Translation termination factor eRF3, has a role in mRNA deadenylation and decay; altered protein conformation creates the [PSI(+)] prion that alters translational fidelity and results in a nonsense suppressor phenotype
tid3-1 (ndc80)	Component of the evolutionarily conserved kinetochore-associated Ndc80 complex; conserved coiled-coil protein involved in chromosome segregation, spindle checkpoint activity, kinetochore assembly and clustering
**50 mM HU**	
**Mutation**	**Gene function**
lst8-6, -15	Component of the TOR signaling pathway
mvd1-1296	ERG19, Mevalonate pyrophosphate decarboxylase, involved in the biosynthesis of isoprenoids and sterols, including ergosterol
nop2-3	Probable RNA m(5)C methyltransferase, essential for processing and maturation of 27S pre-rRNA and large ribosomal subunit biogenesis; localized to the nucleolus; constituent of 66S pre-ribosomal particles
tsc3-2	Protein involved in sphingolipid biosynthesis
**100 mM HU**	
**Mutation**	**Gene function**
act1-111	Actin, structural protein involved in multiple cytoskeletal functions
arp3-G302Y, -H161A, -G15C	Component of the Arp2/3 complex, a highly conserved actin nucleation center required for the motility and integrity of actin patches
cdc2-1	DNA polymerase delta
cdc8-1	Thymidylate and uridylate kinase, de novo biosynthesis of pyrimidine deoxyribonucleotides; converts dTMP to dTDP and dUMP to dUTP
cks1-35	Cyclin-dependent protein kinase regulatory subunit and adaptor
esa1-1851	Catalytic subunit of the histone acetyltransferase complex (NuA4)
gpi8-ts	Subunit of the glycosylphosphatidylinositol transamidase complex
las17-13	Actin assembly factor, activates the Arp2/3 protein complex
mps3-7	Nuclear envelope protein required for SPB duplication and nuclear fusion
nse1-16	Component of the SMC5-SMC6 complex
nse4-ts2	Component of the SMC5-SMC6 complex
pob3-Q308K	Subunit of the heterodimeric FACT complex (Spt16p-Pob3p)
pre2-2	Beta 5 subunit of the 20S proteasome
rfa1-M2	Subunit of heterotrimeric Replication Protein A that binds ssDNA
