# Supplementary material for: Chemical Suppression of Defects in Mitotic Spindle Assembly, Redox Control, and Sterol Biosynthesis by Hydroxyurea
Source: G3 (Bethesda). 2013 Nov 5;4(1):39–48. doi: 10.1534/g3.113.009100 (PMC3887538; doi:10.1534/g3.113.009100)
Supplement: Supporting Information [file supp_g3.113.009100_FigureS1.pdf]

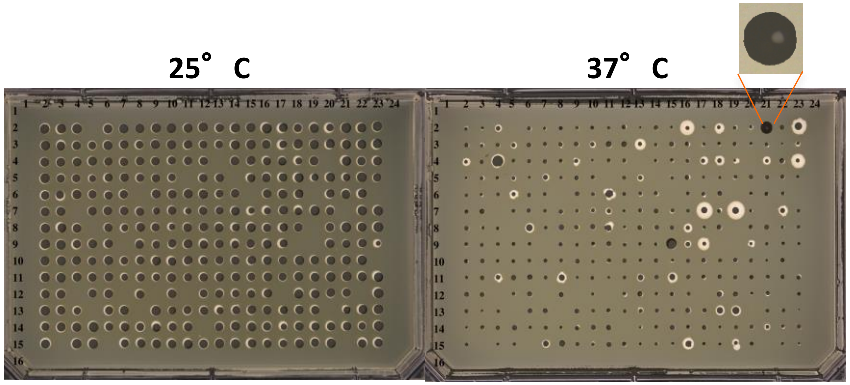

Figure S1 Representative overlaid images of a primary "Chemical Suppression" screen. Cells that were grown in the absence and presence of 50 mM HU were artificially colored white and black, respectively. The left and right images correspond to plates incubated at 25°C and 37°C, respectively. The majority of the temperature-sensitive mutant collection demonstrated temperature-sensitivity at 37°C while a small percentage showed either moderate or no temperature-sensitivity (comparing the white colonies at 25°C and 37°C). We believe that some of these strains might have accumulated suppressor mutations or are revertants. Those cells that showed enhanced growth in the presence of HU at 37°C appear as a grey spot with a black halo, such as *ero1-1* located at row 2, column 21 (inset). The plates were photographed after 3 days growth.
