# Supplementary material for: Chemical Suppression of Defects in Mitotic Spindle Assembly, Redox Control, and Sterol Biosynthesis by Hydroxyurea
Source: G3 (Bethesda). 2013 Nov 5;4(1):39–48. doi: 10.1534/g3.113.009100 (PMC3887538; doi:10.1534/g3.113.009100)
Supplement: Supporting Information [file supp_g3.113.009100_FigureS2.pdf]

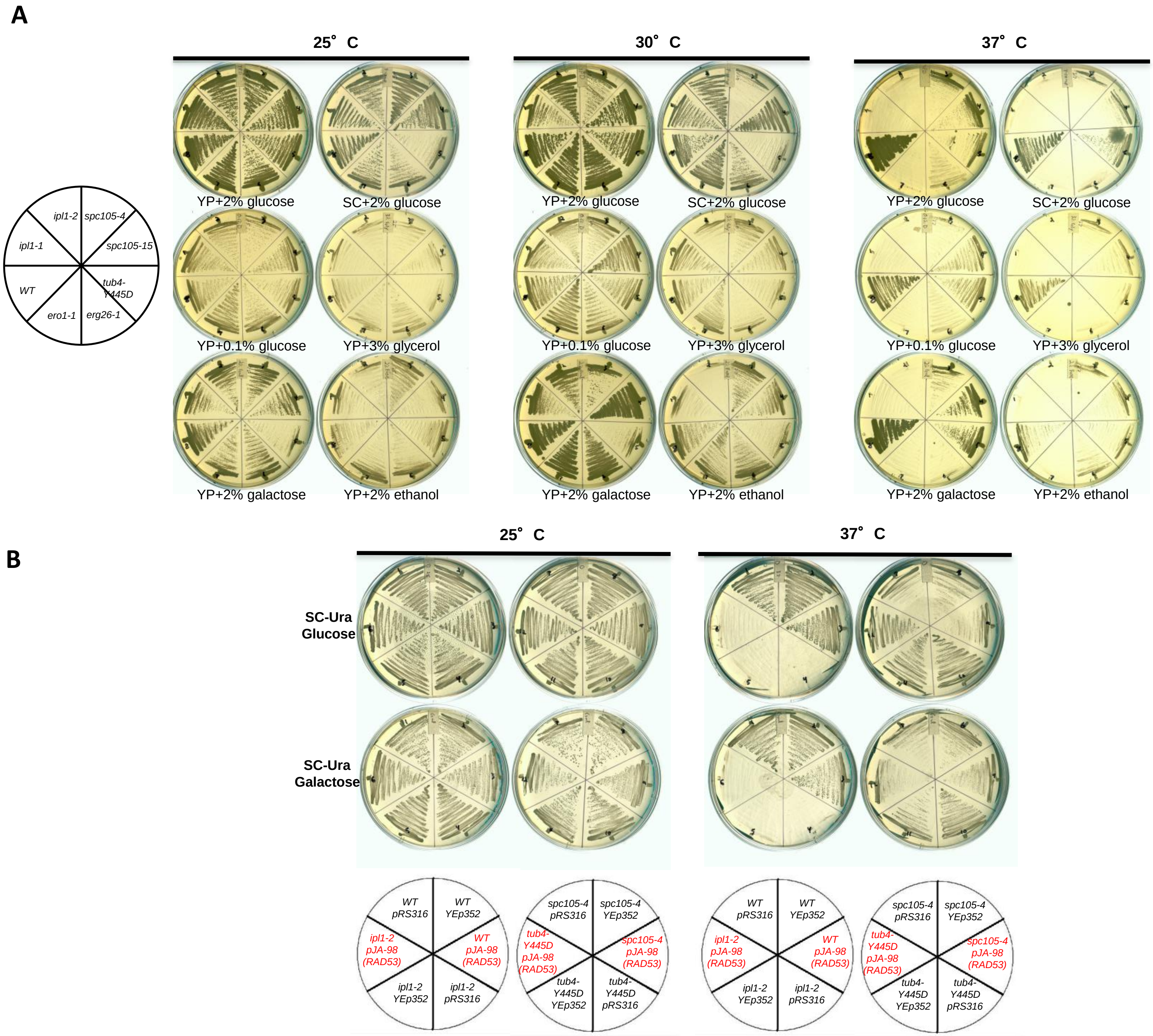
A
25° C
30° C
37° C
ipl1-2
spc105-4
ipl1-1
spc105-15
WT
tub4-Y445D
ero1-1
erg26-1
YP+2% glucose
SC+2% glucose
YP+0.1% glucose
YP+3% glycerol
YP+2% galactose
YP+2% ethanol
B
25° C
37° C
SC-Ura Glucose
SC-Ura Galactose
WT pRS316
WT YEp352
ipl1-2 pJA-98 (RAD53)
WT pJA-98 (RAD53)
ipl1-2 YEp352
ipl1-2 pRS316
spc105-4 pRS316
spc105-4 YEp352
tub4-Y445D pJA-98 (RAD53)
spc105-4 pJA-98 (RAD53)
tub4-Y445D YEp352
tub4-Y445D pRS316

Figure S2 (**A**) The majority of the mutants whose temperature-sensitivity was rescued by HU cannot be rescued by nutrient limitation-induced cell cycle delay. Growth media with different carbon sources, identical in each of the three panels under different temperatures, are as shown. Plates were photographed after three days incubation at the respective temperatures. The order of strains on each plate is indicated by the key on the left. (**B**) Overexpression of the Rad53 kinase does not rescue the temperature sensitivity of the mutants suppressed by HU. WT, *ipl1-2, spc105-4,* and *tub4-Y445D* strains were transformed with the pRS316, YEp352, or pJA-98 (GAL-RAD53) plasmids bearing *URA3* prototrophic marker. pRS316 and YEp352 are empty plasmid controls for the pJA-98(GAL-RAD53) plasmid. The resulting transformants were streaked on SC medium lacking uracil with either 2% glucose (repressed for *RAD53* expression) or 3% galactose (induced for *RAD53* expression). The plates were incubated at either 25°C or 37°C for two days before photographing. The keys to the strains on each plate are shown at the bottom of the panel.
