# Supplementary material for: Chemical Suppression of Defects in Mitotic Spindle Assembly, Redox Control, and Sterol Biosynthesis by Hydroxyurea
Source: G3 (Bethesda). 2013 Nov 5;4(1):39–48. doi: 10.1534/g3.113.009100 (PMC3887538; doi:10.1534/g3.113.009100)
Supplement: Supporting Information [file supp_g3.113.009100_FigureS3.pdf]

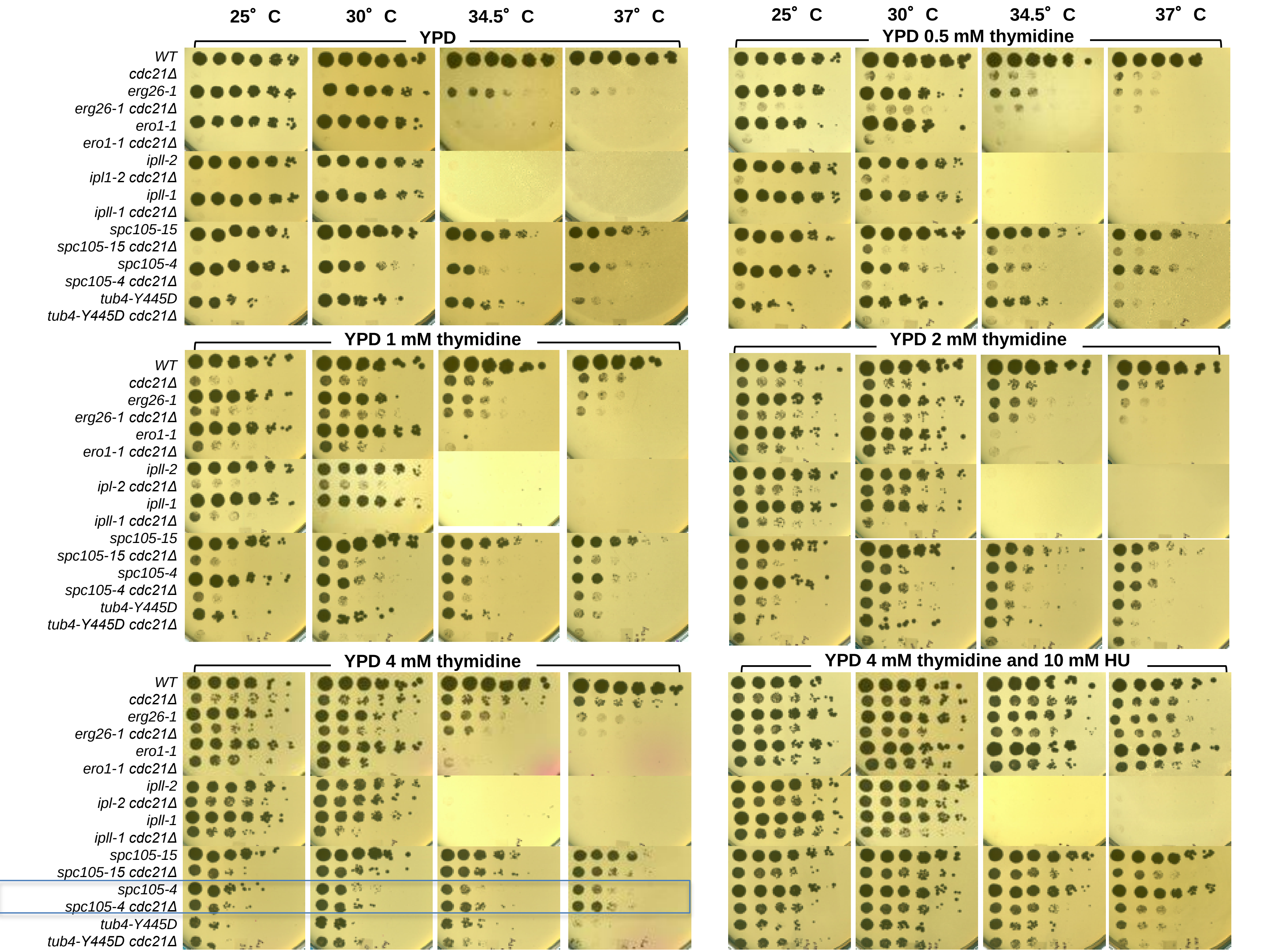

25° C
30° C
34.5° C
37° C
YPD
YPD 0.5 mM thymidine
YPD 1 mM thymidine
YPD 2 mM thymidine
YPD 4 mM thymidine
YPD 4 mM thymidine and 10 mM HU
WT
cdc21Δ
erg26-1
erg26-1 cdc21Δ
ero1-1
ero1-1 cdc21Δ
ipll-2
ipl1-2 cdc21Δ
ipll-1
ipll-1 cdc21Δ
spc105-15
spc105-15 cdc21Δ
spc105-4
spc105-4 cdc21Δ
tub4-Y445D
tub4-Y445D cdc21Δ
ipl-2 cdc21Δ

Figure S3 Thymidine limitation does not suppress most of the temperature-sensitive alleles that are suppressed by HU. Serial diluted cells were spotted on YPD media containing the indicated concentrations of thymidine and incubated at the indicated temperatures for 2-3 days before photographing. YPD medium containing 4 mM thymidine and 10 mM HU served as a control.
