# Supplementary material for: Chemical Suppression of Defects in Mitotic Spindle Assembly, Redox Control, and Sterol Biosynthesis by Hydroxyurea
Source: G3 (Bethesda). 2013 Nov 5;4(1):39–48. doi: 10.1534/g3.113.009100 (PMC3887538; doi:10.1534/g3.113.009100)
Supplement: Supporting Information [file supp_g3.113.009100_FigureS4.pdf]

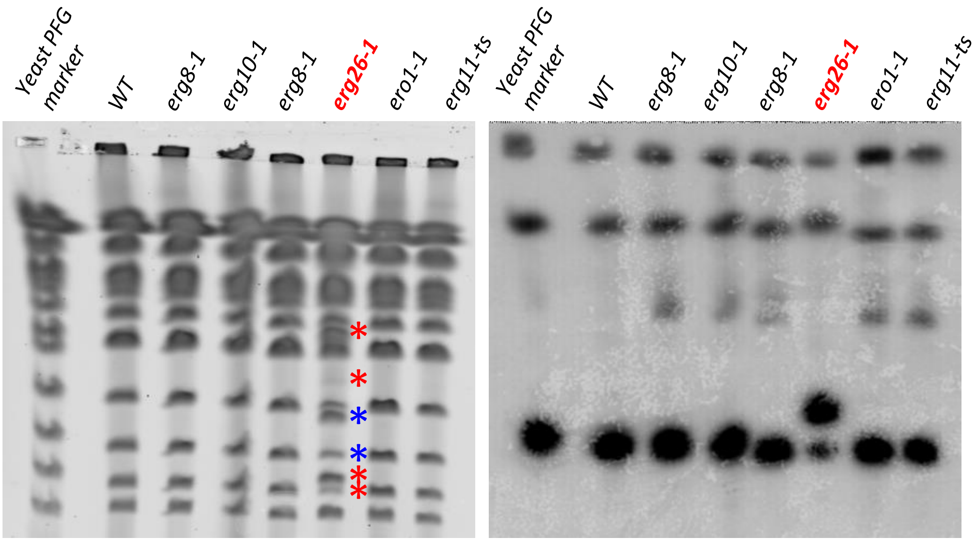

Figure S4 The original *erg26-1* strain from the TS collection exhibits gross chromosomal rearrangement. (**A**) Pulse Field Gel Electrophoresis of yeast chromosomes isolated from different temperature-sensitive strains in the collection. Those chromosome bands that show anomaly such as intensity gain, loss or different size from the control are marked by an asterisk. The blue asterisks indicate the chromosome III species recognized by a mixture of ARS301 and ARS319 probes in (B). (**B**) Southern hybridization of the gel in (A) using a mixture of ARS301 and ARS319 probes.
