# Supplementary material for: Chemical Suppression of Defects in Mitotic Spindle Assembly, Redox Control, and Sterol Biosynthesis by Hydroxyurea
Source: G3 (Bethesda). 2013 Nov 5;4(1):39–48. doi: 10.1534/g3.113.009100 (PMC3887538; doi:10.1534/g3.113.009100)
Supplement: Supporting Information [file supp_g3.113.009100_FigureS5.pdf]

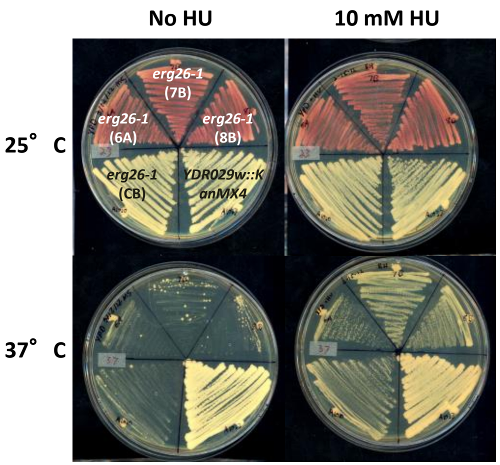

Figure S5 The three isolates of *erg26-1* in W303 (6A, 7B and 8B) are also temperature-sensitive at 37°C and the temperature-sensitivity can be suppressed by 10 mM HU. Cells were streaked on solid YPD medium containing 200 µg/ml G418 with or without HU. A strain containing the replacement of a dubious ORF YDR029w by the KanMX4 cassette and the *erg26-1* strain from the TS collection, *erg26-1* (CB), both in the BY4741 background, served as controls.
