# Supplementary material for: Chemical Suppression of Defects in Mitotic Spindle Assembly, Redox Control, and Sterol Biosynthesis by Hydroxyurea
Source: G3 (Bethesda). 2013 Nov 5;4(1):39–48. doi: 10.1534/g3.113.009100 (PMC3887538; doi:10.1534/g3.113.009100)
Supplement: Supporting Information [file supp_g3.113.009100_FigureS6.pdf]

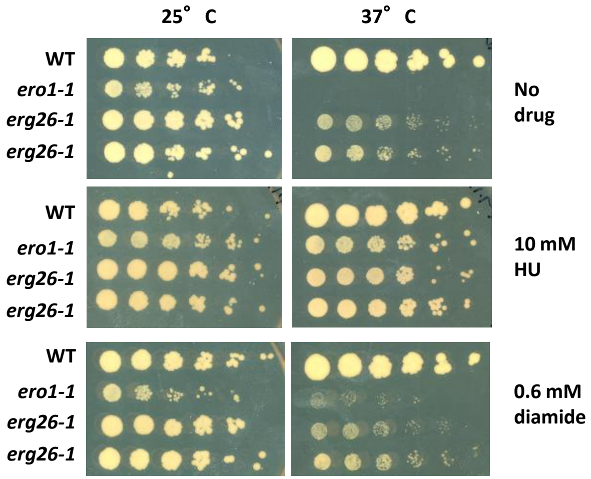

Figure S6 Temperature-sensitivity of *ero1-1* but not *erg26-1* cells can be partially suppressed by the oxidizing chemical diamide. Serial dilutions (1:5) of *ero1-1* and *erg26-1* cells were spotted on solid YPD media containing no drug, or 10 mM HU, or 0.6 mM diamide and incubated at the indicated temperatures for two to three days before photographing.
