# Supplementary material for: Chemical Suppression of Defects in Mitotic Spindle Assembly, Redox Control, and Sterol Biosynthesis by Hydroxyurea
Source: G3 (Bethesda). 2013 Nov 5;4(1):39–48. doi: 10.1534/g3.113.009100 (PMC3887538; doi:10.1534/g3.113.009100)
Supplement: Supporting Information [file supp_g3.113.009100_FigureS7.pdf]

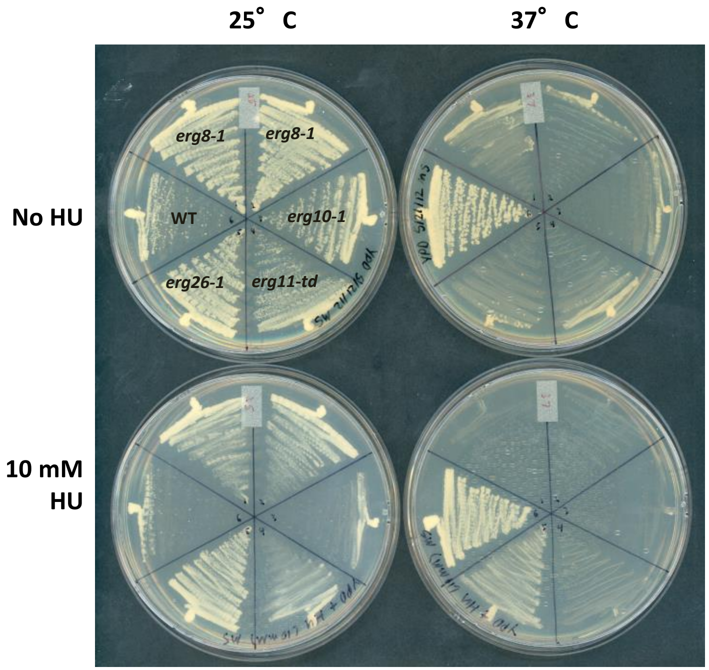

Figure S7 HU specifically suppresses *erg26-1,* not other ergosterol mutants in the temperature-sensitive strain collection. The indicated strains were streaked on YPD medium with or without 10 mM HU and incubated at the indicated temperatures for two to three days before photographing.
