# Supplementary material for: Chemical Suppression of Defects in Mitotic Spindle Assembly, Redox Control, and Sterol Biosynthesis by Hydroxyurea
Source: G3 (Bethesda). 2013 Nov 5;4(1):39–48. doi: 10.1534/g3.113.009100 (PMC3887538; doi:10.1534/g3.113.009100)
Supplement: Supporting Information [file supp_g3.113.009100_TableS1.pdf]

Table S1 Sequence of PCR and sequencing primers for *ERO1* and *ERG26*.

**PCR primers**	
ERG26-F1	5’-gcaactctaccggaagggcaaac-3’
ERG26-R2	5’-gacccggcggggacgaggcaagctaaacagatctattaccccgacgcttcatagtgtagtc-3’
ERO1-F1	5’-acgatacggagtacgtgtcataaaaacttg-3’
ERO1-R2	5’-gtgacccggcggggacgaggcaagctaaacagatctattgatatggaagggtctatttagcggtgt-3’
**Sequencing primers**	
ERG26-seq-R1	5’-cggtgcgtatcagatatgc-3’
ERG26-seq-R2	5’-ggacgaagagcaacagtatag-3’
ERO1-seq-F1	5’-tatggtggtaagcaagctgg-3’
ERO1-seq-F2	5’-caacgacgctgatgaattc-3’
ERO1-seq-R2	5’-gtttgtcactctatcagg-3’
