# Supplementary material for: Chemical Suppression of Defects in Mitotic Spindle Assembly, Redox Control, and Sterol Biosynthesis by Hydroxyurea
Source: G3 (Bethesda). 2013 Nov 5;4(1):39–48. doi: 10.1534/g3.113.009100 (PMC3887538; doi:10.1534/g3.113.009100)
Supplement: Supporting Information [file supp_g3.113.009100_TableS2.pdf]

Table S2 List of mutations that show hypersensitivity to HU.

10 mM HU	
**Mutation**	**Gene function**
prp9-ts	Subunit of the SF3a splicing factor complex, required for spliceosome assembly; acts after the formation of the U1 snRNP-pre-mRNA complex
sup35-td	Translation termination factor eRF3, has a role in mRNA deadenylation and decay; altered protein conformation creates the [PSI(+)] prion that alters translational fidelity and results in a nonsense suppressor phenotype
tid3-1 (ndc80)	Component of the evolutionarily conserved kinetochore-associated Ndc80 complex; conserved coiled-coil protein involved in chromosome segregation, spindle checkpoint activity, kinetochore assembly and clustering
**50 mM HU**	
**Mutation**	**Gene function**
lst8-6, -15	Component of the TOR signaling pathway
mvd1-1296	ERG19, Mevalonate pyrophosphate decarboxylase, involved in the biosynthesis of isoprenoids and sterols, including ergosterol
nop2-3	Probable RNA m(5)C methyltransferase, essential for processing and maturation of 27S pre-rRNA and large ribosomal subunit biogenesis; localized to the nucleolus; constituent of 66S pre-ribosomal particles
tsc3-2	Protein involved in sphingolipid biosynthesis
**100 mM HU**	
**Mutation**	**Gene function**
act1-111	Actin, structural protein involved in multiple cytoskeletal functions
arp3-G302Y, -H161A, -G15C	Component of the Arp2/3 complex, a highly conserved actin nucleation center required for the motility and integrity of actin patches
cdc2-1	DNA polymerase delta
cdc8-1	Thymidylate and uridylate kinase, de novo biosynthesis of pyrimidine deoxyribonucleotides; converts dTMP to dTDP and dUMP to dUTP
cks1-35	Cyclin-dependent protein kinase regulatory subunit and adaptor
esa1-1851	Catalytic subunit of the histone acetyltransferase complex (NuA4)
gpi8-ts	Subunit of the glycosylphosphatidylinositol transamidase complex
las17-13	Actin assembly factor, activates the Arp2/3 protein complex
mps3-7	Nuclear envelope protein required for SPB duplication and nuclear fusion
nse1-16	Component of the SMC5-SMC6 complex
nse4-ts2	Component of the SMC5-SMC6 complex
pob3-Q308K	Subunit of the heterodimeric FACT complex (Spt16p-Pob3p)
pre2-2	Beta 5 subunit of the 20S proteasome
rfa1-M2	Subunit of heterotrimeric Replication Protein A that binds ssDNA
